# Peer Coaching to Support Weight Management in Primary Care: A Cluster Randomized Clinical Trial

**DOI:** 10.1001/jamanetworkopen.2025.29136

**Published:** 2025-09-02

**Authors:** Sandra Wittleder, Laura Wong, Andrea M. Ruan, Nicholas Illenberger, Andrew Nicholson, Dilara Portelli, Rachel Cansler, Craig T. Tenner, Scott E. Sherman, Adrian D. Aguilar, Victoria Sweat, Stephanie L. Orstad, Sarvenaz Vandyousefi, Marina Fronshtein, Shea Smith, Alia Dixon, Michael G. Goldstein, Susan D. Raffa, Judith Wylie-Rosett, Melanie Jay

**Affiliations:** 1 Department of Medicine, Veterans Affairs New York Harbor Healthcare System, New York; 2 Department of Medicine, New York University (NYU) Grossman School of Medicine, New York; 3 Department of Population Health, NYU Grossman School of Medicine, New York; 4 Department of Medicine, Downstate Medical Center, Brooklyn, New York; 5 Veterans Health Administration National Center for Health Promotion and Disease Prevention, Durham, North Carolina; 6 Department of Medical Science, Alpert Medical School, Brown University, Providence, Rhode Island; 7 Department of Medicine, Albert Einstein College of Medicine, Bronx, New York

## Abstract

**Importance:**

Employing peer coaches to provide weight management counseling in primary care could address patient- and clinician-level barriers to obesity care, improve outcomes, and boost engagement in weight management programs.

**Objective:**

To evaluate the efficacy of peer coaching to deliver a low- to moderate-intensity intervention for weight management compared with enhanced usual care (EUC).

**Design, Setting, and Participants:**

This 2-arm, cluster randomized clinical trial was conducted from November 2017 to August 2021 at a single US Department of Veteran Affairs (VA) site. Primary care physicians (PCPs) and their patients with a body mass index (calculated as weight in kilograms divided by height in meters squared) of at least 25 (overweight) or 30 or more (obesity) were invited to participate. Enrollment was stopped early due to the COVID-19 pandemic, when weight management services at the VA site transitioned to primarily virtual care. PCPs were randomized to either the peer coaching (ie, Peer-Assisted Lifestyle) intervention or EUC arm, and their patients received the corresponding treatment for those arms. Data were analyzed according to the intention-to-treat principle from February 2023 and July 2024.

**Interventions:**

Patients in the peer coaching arm completed a tablet-based, goal-setting tool and received 1 in-person and up to 12 individual peer-coaching telephone sessions over 1 year. Peer coaches were veterans with a bachelor’s degree and training for a minimum of 20 hours. Patients in the EUC control arm received health education materials.

**Main Outcomes and Measures:**

The primary outcome was mean (SE) change in weight (in kilograms) at 12 months. Secondary outcomes included mean (SE) weight change in percentage, proportion of patients achieving 5% or higher weight loss, and change in waist circumference in inches.

**Results:**

A total of 20 PCPs (11 women [55.0%]) and 281 patients were enrolled. Patients had a mean (SD) age of 50.6 (11.5) years and included 221 men (78.6%). The mean (SD) body mass index at baseline was 33.4 (5.1). At 12 months, the adjusted mean (SE) weight change was −2.51 (0.73) kg in the peer coaching arm and −0.79 (0.48) kg in the EUC arm, but the difference was not statistically signficant (difference, −1.72 [0.88] kg; *P* = .05). At 6 months, the adjusted mean (SE) proportion of patients who lost at least 5% of body weight was 16.68% (0.47%) in the peer coaching arm vs 5.50% (0.32%) in the EUC arm (difference, 11.18 [5.22] percentage points; *P* = .03). At 6 months, the adjusted mean (SE) proportion of patients who attended a weight management program was 28.68% (5.37%) in the peer coaching arm and 13.32% (3.38%) in the EUC arm (difference, 15.37 [6.45] percentage points; *P* = .02).

**Conclusions and Relevance:**

In this randomized clinical trial, a low- to moderate-intensity peer-coaching intervention did not result in greater weight loss at 12 months, but it improved attendance at weight management programs.

**Trial Registration:**

ClinicalTrials.gov Identifier: NCT03163264

## Key Points

**Question:**

Can a primary care-based peer coaching program lead to greater weight loss than enhanced usual care (EUC)?

**Findings:**

In this cluster randomized clinical trial involving 20 primary care physicians and 281 of their patients, there was no difference in weight loss between groups at 12 months; however, patients in the peer coaching group lost a higher percentage of weight at 12 months and were more likely to join a weight loss program at 6 months.

**Meaning:**

The findings suggest that peer coaching can produce weight loss in primary care and promote enrollment in a weight management program.

## Teaser

This randomized clinical trial assesses the effect of a health-coaching intervention on weight loss among veterans at a Veteran Affairs health care system.

## Introduction

Obesity is a major contributor to chronic disease morbidity and mortality.^1,2^ A 2014 study reported that 78% of US veterans receiving care in the Veterans Health Administration had a body mass index (BMI; calculated as weight in kilograms divided by height in meters squared) in the overweight (≥25) or obesity range (≥30), with 41% classified as having obesity.^3^ Obesity rates have continued to increase.^4^ Veterans often face socioeconomic challenges, and many veterans belong to racially and ethnically minoritized groups, which are associated with a higher obesity burden^5,6^ and a lower response to lifestyle changes, medications, or surgery compared with the general population.^7,8,9^ Further research is needed to improve treatment responses in these populations.

With more than 480 million primary care visits annually in the US,^10^ integrating obesity treatment into primary care could have a substantial impact on public health. The US Preventive Services Task Force recommends that clinicians offer or refer adults with obesity to intensive, multicomponent behavioral interventions, as these interventions produce clinically significant (≥5%) weight loss.^11^ However, only 40% of adults with overweight or obesity reported receiving weight counseling,^12^ and less than 8% of eligible veterans attended the US Department of Veterans Affairs (VA) MOVE! Weight Management Program, an evidence-based comprehensive lifestyle intervention that focuses on nutrition and physical activity changes supported by behavioral strategies to help veterans improve their health through weight management.^13,14,15^

Low rates of weight management counseling and intensive program attendance can be attributed to several physician- and patient-level barriers. Physicians often lack sufficient training and time.^16,17,18^ Patient-level barriers include scheduling challenges and competing responsibilities, such as caregiving and work, and prior unfavorable experiences with weight-related stigma^19^ in health care settings, which disproportionately affect socioeconomically disadvantaged groups.^20,21^ Peer coaches—nonclinical laypersons with shared backgrounds or experiences—can help mitigate these barriers. Peer coaches are uniquely positioned to offer culturally relevant support and behavior change counseling outside of constrained clinic visits.^22^ Given their shared military experience, peer coaches may better understand and address barriers specific to veterans, such as the transition from a highly structured military environment to civilian life.^23^ As patients, veterans may feel more comfortable speaking with peer coaches rather than health care professionals, which can help address weight-related stigma.^24,25^ Evidence supports the efficacy of peer-delivered interventions across chronic health conditions, including obesity.^26,27,28,29,30^

To address patient- and clinician-level barriers to weight management, we conducted a 2-arm cluster randomized clinical trial comparing a peer-coaching intervention (ie, the Peer-Assisted Lifestyle) with enhanced usual care (EUC) at the VA. The peer-coaching intervention uses low-cost technology and peer coaches to provide low- to moderate-intensity, one-to-one counseling with flexible patient-centered scheduling while encouraging participation in more intensive weight management programs. We hypothesized that patients of primary care physicians (PCPs) randomly assigned to the peer coaching intervention arm would experience greater weight loss than those in the EUC arm at 12 months.

## Methods

### Study Design and Randomization

This cluster randomized clinical trial aimed to evaluate the efficacy of peer coaching compared with EUC to deliver a low- to moderate-intensity intervention for weight management. PCPs were randomized to the peer coaching intervention arm or the EUC arm in a 1:1 ratio using a random number generator (Figure). Eligible enrolled patients received the intervention assigned to their PCPs (cluster). Investigators who participated in the data analyses and interpretation were blinded to patient and PCP assignments. The trial was approved by the institutional review board at the VA New York Harbor Healthcare System; the trial protocol is provided in Supplement 1. All participants provided written informed consent. The methods have been published previously.^31^ We followed the Consolidated Standards of Reporting Trials (CONSORT) reporting guideline.

**Figure.**
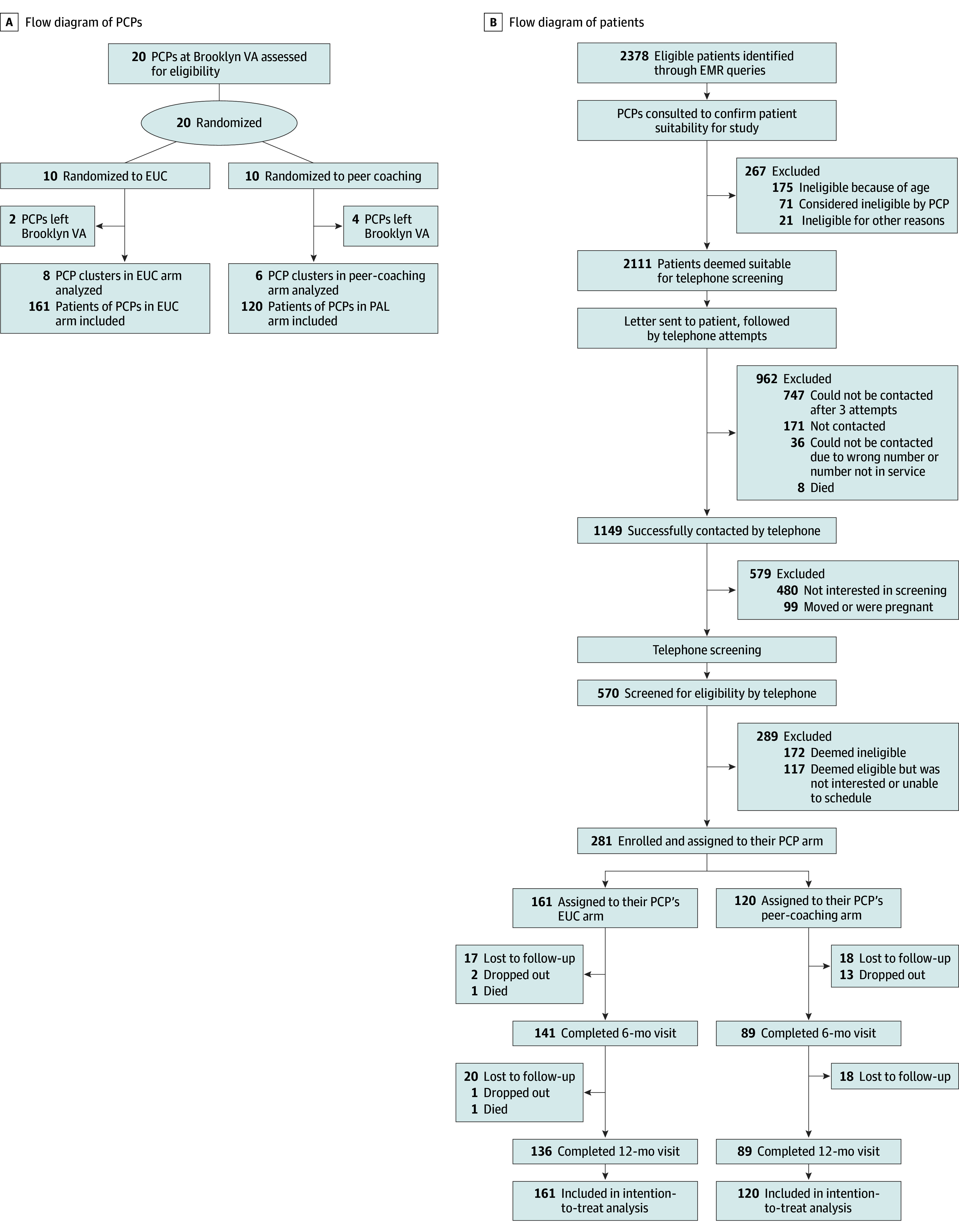
CONSORT Flow Diagram of the Cluster Randomization of Primary Care Providers (PCPs) and Patients Brooklyn VA indicates Brooklyn campus of the Veterans Affairs New York Harbor Healthcare System; EMR, electronic medical record; and EUC, enhanced usual care.

### Participants and Setting

The study was conducted from November 2017 to August 2021 in primary care clinics at the Brooklyn campus of the VA New York Harbor Healthcare System (hereafter Brooklyn VA). PCPs were eligible if their panel consisted of 250 patients or more. Patients were eligible if they were 18 to 69 years of age and had a BMI of 30 or higher (indicating obesity) or 25 or higher (indicating overweight) with a weight-associated comorbidity. The full inclusion and exclusion criteria and recruitment procedures are provided in eMethods 1 and 2 in Supplement 2.

### Treatment Arms

Patients in the EUC (control) arm were provided publicly available handouts (part of VA’s Healthy Living messages^32^) by research assistants, along with information regarding the MOVE! program. Patients were instructed to subsequently follow up with their PCP for usual care.

The peer coaching intervention was designed to integrate weight management counseling in primary care using the 5As framework (Assess, Advise, Agree, Assist, and Arrange),^33^ which included providing initial low- to moderate-intensity peer counseling as well as supporting enrollment and engagement in MOVE! or other intensive weight management programs. Patients were assigned to peer coaches based on mutual availability. Peer coaches were veterans who had a bachelor’s degree; were recruited via an online job posting; and were trained for a minimum of 20 hours using role-playing, shadowing, and listening to recordings. Patients received 1 in-person counseling session and up to 12 telephone calls. Before the first coaching session, patients used a self-guided, tablet-based, goal-setting tool that assessed current behaviors and barriers (Assess), generated personalized feedback (Advise), and produced personalized health behavior reports and educational handouts, which formed the basis for collaborative goal-setting using motivational interviewing and SMART (Specific, Measurable, Attainable, Relevant, Timely) goal setting during the initial session (Agree). Patients then received up to 12 telephone-based coaching calls. Calls reinforced goal adherence, encouraged incremental behavior changes, and addressed barriers to care and social determinants of health (eg, connecting patients to services to address food insecurity) based on a coaching toolkit (Assist). Additionally, the tablet application generated documentation templates for the peer coach to upload to the VA electronic health record, supporting care continuity and communication with PCPs (Arrange). Details of the intervention, including training and fidelity, are included in eMethods 3 in Supplement 2.

### Data Collection

Research assistants (L.W., A.M.R., D.P., and S.S.) collected clinical measures (weight, height, waist circumference, and blood pressure) and survey data (demographics, weight management program attendance, physical activity, and dietary outcomes) during in-person study visits at baseline, 6 months, and 12 months. Race and ethnicity were self-reported by participants and included the following categories: Hispanic, non-Hispanic Black (hereafter Black), non-Hispanic White (hereafter White), and non-Hispanic other (including Aboriginal, American Indian or Alaska Native; Asian; Celtic, Ecuadorian, Jewish, Native Hawaiian or Other Pacific Islander, Panamanian, West Asian, and West Indian). These data were included in this trial to describe the sample in terms of representativeness of the population and to explore differences in weight loss trajectories across groups.

Detailed descriptions of the clinical and behavioral outcome measures and procedures, including COVID-19 protocol changes, are included in eMethods 4 in Supplement 2. All data were entered into REDCap 8.1.11 (National Institutes of Health). A comprehensive list of measures assessed in the trial is available elsewhere.^31^

### Statistical Analysis

Details of the sample size calculation are included in eMethods 5 in Supplement 2. Data were analyzed between February 2023 and July 2024. The primary outcome was the mean (SE) weight change in kilograms at 12 months. Secondary clinical outcomes included mean (SE) weight change in percentage, proportion of patients achieving 5% or higher weight loss, and waist circumference change in inches. Other secondary outcomes included weight management program attendance and changes in physical activity and diet.

Differences between the peer coaching and EUC arms on each outcome were assessed at the 6- and 12-month follow-up. Primary analytic results used a mixed-effects regression modeling approach. Regression analyses accounted for the correlation between patients seen by the PCPs using a random intercept. We adjusted for baseline characteristics that differed between arms, such as height and fruit and vegetable intake, which was assessed using the 6-item subscale of the Food Behavior Checklist. Scores could range from 1 to 4, with higher scores indicating more or healthier fruit and vegetables intake. In addition, due to a lapse in enrollment in the peer coaching arm between August and December 2018 related to the attrition of both peer coaches, a greater proportion of patients in this arm were enrolled later and completed follow-up after the COVID-19 outbreak. Time of enrollment (number of days from first enrollment) was adjusted to account for potential time effects. For analyses using mixed-effects regression models, we used a multiple imputation approach for missing data under the assumption that data were missing at random (Supplement 1). Fifty datasets were imputed using a Monte Carlo approach with chained equations. Imputations were performed separately for the peer coaching and EUC arms to permit greater flexibility. Exploratory analyses were conducted to identify patient characteristics and intervention components that may have influenced intervention effectiveness. These analyses are described in eMethods 6 in Supplement 2.

All statistical tests were 2-tailed, with a *P* < .05 significance level, and followed the intention-to-treat principle. Analyses were performed using R, version 3.14.0 (R Project for Statistical Computing).^34^

## Results

Twenty PCPs (11 women [55.0%], 9 men [45.0%]; mean [SD] years of VA service, 13.6 [12.0] years) were invited to participate. All 20 PCPs were eligible, agreed to participate, and were randomly assigned to either the peer coaching or EUC arm; 6 PCPs left the study. Additionally, 2378 patients were recruited, of whom 281 were enrolled. The Figure shows the flowcharts for PCPs and patients.

Table 1 shows the sociodemographic and health characteristics of the 281 enrolled patients, which included 60 women (21.4%) and 221 men (78.6%), with a mean (SD) age of 50.6 (11.5) years. Of these patients, 61 (21.7%) identified as Hispanic, 148 (52.7%) as Black, 48 (17.1%) as White, and 24 (8.5%) as other race and ethnicity. Patients had a mean (SD) BMI of 33.4 (5.1), and waist circumference of 42.8 (5.4) inches. Except for mean (SD) height (173.8 [8.5] cm vs 171.5 [8.9] cm) and fruit and vegetable intake scores (2.3 [1.3] vs 2.6 [1.2]), there were no other significant differences in baseline characteristics between EUC and peer coaching arms (Table 1). There was no significant evidence of a differential rate of pre- or post-COVID-19 outbreak observations between the peer coaching and EUC arms at 6 months (39 [32.5%] vs 44 [27.3%]; *P* = .42). However, there was evidence of a differential rate at 12 months between the peer coaching and EUC arms after March 2020 (87 [72.5%] vs 59 [36.6%]; *P* < .001).

**Table 1.** Baseline Characteristics of Veteran Patients

Characteristic	Patients, No. (%)		
	Overall (N = 281)^a^	EUC (n = 161)	Peer coaching (n = 120)
Age, mean (SD), y	50.6 (11.5)	49.9 (11.6)	51.6 (11.3)
Gender			
Women	60 (21.4)	29 (18.0)	31 (25.8)
Men	221 (78.6)	132 (82.0)	89 (74.2)
Race and ethnicity^b^			
Hispanic	61 (21.7)	38 (23.6)	23 (19.2)
Non-Hispanic Black	148 (52.7)	87 (54.0)	61 (50.8)
Non-Hispanic White	48 (17.1)	26 (16.1)	22 (18.3)
Non-Hispanic other^b^	24 (8.5)	10 (6.2)	14 (11.7)
Born in US			
Yes	194 (69.0)	110 (68.3)	84 (70.0)
No	87 (31.0)	51 (31.7)	36 (30.0)
English at home			
Yes	262 (93.9)	150 (93.8)	112 (94.1)
No	17 (6.1)	10 (6.2)	7 (5.9)
Employment status^c^			
Employed	159 (56.8)	98 (61.3)	61 (50.8)
Unemployed	121 (43.2)	62 (38.8)	59 (49.2)
Marital status^d^			
Married or in a marriage-like relationship	158 (56.2)	91 (56.5)	67 (55.8)
Single, never married, separated, divorced, or widowed	123 (43.8)	70 (43.5)	53 (44.2)
Educational level^e^			
≤High school diploma	58 (20.6)	30 (18.6)	28 (23.3)
>High school diploma	223 (79.4)	131 (81.4)	92 (76.7)
Depression risk: CES-D-SF score ≥8^f^			
Yes	68 (24.3)	35 (21.7)	33 (27.7)
No	212 (75.7)	126 (78.3)	86 (72.3)
Clinical and behavioral measures			
Height, mean (SD), cm	172.8 (8.8)	173.8 (8.5)	171.5 (8.9)
Weight, mean (SD), kg	99.9 (18.2)	100.6 (16.4)	98.9 (20.4)
BMI, mean (SD)	33.4 (5.1)	33.3 (4.7)	33.5 (5.6)
Waist circumference, mean (SD), in	42.8 (5.4)	42.7 (4.7)	42.9 (6.2)
Diastolic blood pressure, mean (SD), mm Hg	78.5 (19.9)	77.8 (10.5)	79.3 (28.0)
Systolic blood pressure, mean (SD), mm Hg	125.6 (33.1)	126.9 (41.9)	123.8 (14.4)
Walking physical activity, mean (SD), min/wk^f^	350.6 (383.3)	364.5 (408.2)	332.0 (347.9)
Moderate physical activity, mean (SD), min/wk^f^	177.1 (278.3)	185.3 (281.5)	166.1 (274.9)
Vigorous physical activity, mean (SD), min/wk^f^	204.0 (284.8)	213.1 (282.4)	191.7 (288.7)
Total physical activity, mean (SD), MET min/wk^g^	3511.0 (3570.1)	3666.6 (3699.1)	3301.7 (3393.3)
Fruit and vegetable intake score, mean (SD)^h^	2.4 (1.3)	2.3 (1.3)	2.6 (1.2)
Healthy dietary behavior score, mean (SD)^i^	13.2 (3.4)	13.1 (3.5)	13.4 (3.4)
Sweet and salty snack intake score, mean (SD)^j^	4.5 (1.2)	4.6 (1.2)	4.4 (1.1)

Abbreviations: BMI, body mass index (calculated as weight in kilograms divided by height in meters squared); CES-D-SF, Center for Epidemiologic Studies Depression Scale Short-Form; EUC, enhanced usual care; MET, metabolic equivalent.

^a^ Percentage missing ranged from 0% to 0.7%.

^b^ Race and ethnicity were self-reported. Other races and ethnicities were combined due to small cell sizes.

^c^ Employed included working full-time and working part-time. Unemployed included unemployed or laid off, looking for work, student, keeping house or raising children full-time, and retired.

^d^ Marital status included single or never married, married or in a marriage-like relationship, separated, divorced, and widowed.

^e^ Educational level included never attended school or only kindergarten, grades 1 through 8, grades 9 through 11, grade 12 or General Educational Development, associate’s degree, some college, college 4 years or more, some graduate or professional training, and graduate or professional degree.

^f^ Walking, moderate, and vigorous durations to maximal 180 minutes and set days with less than 10 minutes of activity to 0, were truncated consistent with the International Physical Activity Questionnaire–Short Form scoring protocol. MET minutes were computed, representing the multiplicative value of energy expended carrying out physical activity greater than 1 MET expended at rest. Walking, moderate, and vigorous MET-minutes per week comprised the continuous total MET-minutes per week score.

^g^ CES-D-SF, a 7-item screener used in US community studies for suspected major depressive disorder (range: 0-21, with higher scores indicating increased likelihood of depression).

^h^ Assessed using the 6-item subscale of the Food Behavior Checklist (range: 1-4, with higher scores indicating more or healthier fruit and vegetables intake).

^i^ Assessed using the 6-item subscale from the Latino Dietary Behaviors Questionnaire (range: 0-24, with higher scores indicating healthier eating behaviors).

^j^ Assessed using 2 items from the Rapid Eating Assessment for Participants Shortened Version (range: 2-6, with higher scores indicating healthier [ie, less frequent] snack intake).

### Changes in Clinical and Behavioral Outcomes

Table 2 shows clinical outcomes assessed at the baseline, 6-month, and 12-month visits. Mean (SE) weight change at 12 months was not significantly different between the peer coaching and EUC arms (−2.51 [0.73] kg vs −0.79 [0.48] kg; difference, −1.72 [0.88] kg; *P* = .05). At 6 months, mean (SE) weight change (−2.10 [0.58] kg vs −0.44 [0.39] kg; difference, −1.66 [0.69] kg; *P* = .02) and percentage weight change (−1.91% [0.59%] vs −0.43% [0.39%]; difference, −1.48 [0.7] percentage points; *P* = .04) was significantly different between the 2 arms. A higher mean (SE) proportion of patients who received the peer coaching intervention lost at least 5% of weight at 6 months compared with patients in the control arm (16.68% [0.47%] vs 5.50% [0.32%]; difference, 11.18 [5.22] percentage points; *P* = .03) (Table 2).

**Table 2.** Adjusted Changes in Clinical Outcomes Between the Treatment Arms at 6 and 12 Months

Outcomes	Adjusted Mean (SE)			*P* value
	EUC	Peer coaching	Difference between peer coaching and EUC	
Weight change at 6 mo, kg	−0.44 (0.39)	−2.10 (0.58)	−1.66 (0.69)	.02
Weight change at 12 mo (primary outcome), kg	−0.79 (0.48)	−2.51 (0.73)	−1.72 (0.88)	.05
Weight change at 6 mo, %	−0.43 (0.39)	−1.91 (0.59)	−1.48 (0.7)	.04
Weight change at 12 mo, %	−0.69 (0.47)	−2.32 (0.75)	−1.63 (0.89)	.07
% With weight loss ≥5% at 6 mo	5.50 (3.16)	16.68 (4.75)	11.18 (5.22)	.03
% With weight loss ≥5% at 12 mo	15.30 (3.38)	26.38 (5.12)	11.07 (6.18)	.07
Waist circumference change at 6 mo, in	−0.72 (0.23)	−1.44 (0.31)	−0.72 (0.39)	.07
Waist circumference change at 12 mo, in	−1.40 (0.29)	−1.82 (0.37)	−0.42 (0.48)	.38

Abbreviation: EUC, enhanced usual care.

Table 3 shows the behavioral outcomes at the baseline, 6-month, and 12-month visits. A higher proportion of patients in the intervention group than the control group reported attending a weight management program, but this was significantly different only at 6 months (28.68% [5.37%] vs 13.32% [3.38%]; difference, 15.37 [6.45] percentage points; *P* = .02). Mean (SE) sweet and salty snack intake scores decreased more in the peer coaching arm than the EUC arm at 6 months (0.60 [0.12] vs 0.24 [0.10]; difference, 0.36 [0.16] percentage points; *P* = .02) and 12 months (0.62 [0.14] vs 0.20 [0.11]; difference, 0.42 [0.18] percentage points; *P* = .02). The peer coaching intervention did not have significant changes in fruit and vegetable intake scores, healthy dietary behavior scores, or physical activity compared with the EUC arm.

**Table 3.** Adjusted Changes in Behavioral Outcomes Between the Treatment Arms at 6 and 12 Months

Outcomes	Mean (SE)			*P* value
	EUC	Peer coaching	Difference between peer coaching and EUC	
Weight management program attendance at 6 mo, %^a^	13.32 (3.38)	28.68 (5.37)	15.37 (6.45)	.02
Weight management program attendance at 12 mo, %^a^	14.44 (3.78)	23.27 (4.39)	8.83 (5.73)	.12
Change in vigorous physical activity at 6 mo, min/wk^b^	50.00 (30.53)	74.82 (40.47)	24.82 (51.72)	.63
Change in vigorous physical activity at 12 mo, min/wk^b^	36.33 (31.47)	0.01 (35.66)	−36.32 (47.71)	.45
Change in moderate physical activity at 6 mo, min/wk^b^	50.51 (27.48)	17.46 (33.32)	−33.05 (44.3)	.46
Change in moderate physical activity at 12 mo, min/wk^b^	9.25 (28.13)	12.21 (34.3)	2.96 (45.05)	.95
Change in walking physical activity at 6 mo, min/wk^b^	18.33 (39.58)	100.95 (49.4)	82.62 (65.47)	.21
Change in walking activity, 12 mo at min/wk^b^	−26.41 (38.25)	−2.67 (45.5)	23.74 (60.19)	.69
Change in total physical activity at 6 mo, MET min/wk^b^	675.10 (327.88)	1012.63 (442.23)	337.53 (568.21)	.55
Change in total physical activity at 12 mo, MET min/wk^b^	244.47 (326.9)	47.37 (379.36)	−197.10 (510.19)	.70
Change in fruit and vegetable intake score at 6 mo^c^	−0.14 (0.12)	−0.12 (0.14)	0.02 (0.18)	.89
Change in fruit and vegetable intake score at 12 mo^c^	−0.32 (0.12)	−0.31 (0.14)	0.01 (0.18)	.96
Change in healthy dietary behaviors score at 6 mo^d^	0.99 (0.57)	1.21 (0.68)	0.22 (0.82)	.78
Change in healthy dietary behaviors score at 12 mo^d^	1.44 (0.58)	1.89 (0.69)	0.45 (0.84)	.59
Change in sweet and salty snack intake score at 6 mo^e^	0.24 (0.10)	0.60 (0.12)	0.36 (0.16)	.02
Change in sweet and salty snack intake score at 12 mo^e^	0.20 (0.11)	0.62 (0.14)	0.42 (0.18)	.02

Abbreviations: EUC, enhanced usual care; MET, metabolic equivalent.

^a^ Attendance in both MOVE! and other programs outside the US Department of Veterans Affairs (VA). Attendance most often reported was for the VA’s MOVE! or Telephone Lifestyle Coaching at 6 months (32 [74.4%]) and at 12 months (29 [74.4%]).

^b^ From the International Physical Activity Questionnaire–Short Form, MET minutes were computed, representing the multiplicative value of energy expended carrying out physical activity greater than 1 MET expended at rest. Walking, moderate, and vigorous MET-minutes per week comprised the continuous total MET-minutes per week score.

^c^ Assessed using the 6-item subscale of the Food Behavior Checklist. Scores could range from 1 to 4, with higher scores indicating more or healthier fruit and vegetables intake.

^d^ Assessed using the 6-item subscale from the Latino Dietary Behaviors Questionnaire (range: 0-24, with higher scores indicating healthier eating behaviors).

^e^ Assessed using 2 items from the Rapid Eating Assessment for Participants Shortened Version (range: 2-6, with higher scores indicating healthier [less frequent] snack intake).

### Exploratory Analyses

Results of the exploratory analyses are shown in the eFigure and eTables 1 to 5 in Supplement 2. Mean (SD) number of completed calls (received and answered) in the peer coaching arm was 4.40 (3.02) out of 12 possible calls. We found that the peer coaching intervention may have had a lesser impact on Black patients than patients from other racial and ethnic groups (eFigure, A in Supplement 2). Women in the EUC arm experienced similar weight loss as patients in the peer coaching arm (eFigure, B in Supplement 2). Patients in the EUC arm who attended a weight management program also experienced similar weight loss as patients in the peer coaching arm (eFigure, D in Supplement 2). Compared with men, women in the EUC arm were more likely to join a weight management program. Among patients in the EUC arm with complete follow-up information, 7 of 18 women (38.9%) reported joining a weight management program at follow-up compared with 15 of 109 men (13.8%). The rate of attendance in the peer coaching arm was similar among women and men (9 of 16 [56.3%] vs 24 of 62 [38.7%]) (eTable 2 in Supplement 2). Our data indicated differences in weight management program attendance by race and ethnicity. Across both arms, 31.8% (35 of 110) of Black patients attended a weight management program compared with 21.1% (20 of 95) of patients from all other racial and ethnic categories combined (eTable 1 in Supplement 2). In the peer coaching arm, patients who received a higher number of calls experienced greater weight loss (eFigure, E in Supplement 2). There were no differences in completed coaching calls by gender or race and ethnicity.

Additional sensitivity analyses were conducted to remove data from patients who received obesity care medications during the study period to evaluate the effect these medications may have had on study conclusions. Results were similar to the primary study conclusions. The per-protocol analysis showed that the peer coaching intervention led to increased mean (SE) weight change at 12 months of follow-up (−2.56 [0.74] kg vs −0.79 [0.48] kg; difference, −1.77 [0.89] kg; *P* = .048) (eTable 4 in Supplement 2).

### Adverse Events

In the peer coaching arm, 41 adverse events were reported, affecting 32 patients (26.7%). In the EUC arm, 60 adverse events were reported, affecting 53 patients (32.9%). None of these events were coded as definitely related, and 1 event was coded as possibly related, which involved back spasms and neck pain during physical activity. None were reported as serious.

## Discussion

This trial found that while patients who received a 5As-based, technology-assisted weight management intervention delivered by veteran peer coaches did not lose statistically significant more weight at 12 months than those in the EUC arm, they showed greater weight loss, both in kilograms and percentage. Additionally, a higher proportion of patients who received peer coaching achieved 5% or greater weight loss and reported attending a weight management program. The mean (SE) weight loss in the peer coaching arm was consistent with expectations and similar to other low- to moderate-intensity interventions used in primary care (1 to 3 kg).^35^ It was also consistent with a systematic review of the MOVE! Weight Management Program, showing −0.13 kg to −3.3 kg weight loss in clinical settings where attendance was 2% to 12%.^36^ Studies of high-intensity health coaching interventions with more frequent interactions, such as the PROPEL study with 23 visits in the first 6 months, have been shown to promote greater weight loss (−7.22 kg [95% CI, −8.25 to −6.19] kg).^37^

This trial contributes to the growing body of literature supporting the use of peer coaches for weight management^38,39,40^ and increasing enrollment in intensive weight management programs, such as MOVE! Leahey and Wing^40^ found that peer coaches performed similarly to professional coaches. However, the peer coaches in that study were also participants in the weight management program, unlike the veteran peer coaches in the present study. It remains unclear whether peer coaches are more effective than non–peer health coaches, but peers may have an advantage due to shared experiences. In this trial’s sister study, Goals for Eating and Moving (GEM), the student health coaches were not peers, and a significant difference in mean weight change was not observed between the intervention (GEM) and control (EUC) arms.^41^

With the peer coaching intervention, we aimed to overcome barriers to enrollment and attendance at weight management programs by having peer coaches provide goal setting, brief motivational interviewing, and a toolkit to address these barriers. We found that patients in the peer coaching arm were more likely to attend a weight management program at 6 months than those in the EUC arm. However, at 12 months, attendance likely decreased due to the COVID-19 pandemic, which paused most programs, including the MOVE! program,^42^ affecting 146 (52.0% [87 in the peer coaching arm; 59 in the EUC arm]) enrolled patients. Additionally, the pandemic created several barriers to lifestyle change and weight management, particularly for racially and ethnically minoritized groups in New York City,^43,44,45^ which may have contributed to the lower than expected weight loss observed in this study.

Further exploratory analyses indicated that women in the EUC arm were more likely to join a weight management program. This finding may explain why women in the EUC arm lost similar amounts of weight as those in the peer coaching arm. While other studies have shown greater weight loss among men than women,^45,46^ our exploratory analyses did not indicate such differences in the peer coaching arm.

Our results also showed that Black patients experienced less weight loss compared with patients of other races and ethnicities. This finding aligns with other trials that highlight disparities in the uptake of and response to evidence-based weight-loss interventions among marginalized groups.^8,9^ Unlike previous research, the smaller weight loss among Black patients in the present study could not be attributed to lower engagement with interventional components.^47,48,49^ Furthermore, we did not observe baseline characteristic differences that could indicate additional barriers. However, there may be unmeasured indicators of social determinants of health that may explain the attenuated weight loss in Black patients. More research is needed to understand the drivers of differential treatment responses, and interventions may need to be tailored specifically for Black patients^19^ to address their unique barriers and enhance weight loss outcomes.

The findings of this study suggest future areas of research. While new, highly effective medications for weight management are more effective than lifestyle behavior change alone,^50^ medication adherence in clinical settings remains low^51^ due to factors such as managing adverse effects and maintaining a healthy lifestyle. Future studies may test the use of peer coaches to support patients on these medications and directly compare the effectiveness of peer vs nonpeer coaches.

### Strengths and Limitations

This trial has several strengths. It involved a diverse patient population, with groups that are typically underrepresented in obesity research. Given that the patients were veterans, the trial had a higher percentage of men than most weight management studies, which typically include 60% to 90% women.^52,53^ Another strength is the integration of peer-health coaches into clinics, a novel and feasible low-cost model for weight management delivery in underresourced primary care settings. It was also innovative that the health coaches promoted the use of existing weight management programs within the VA system.

This study has limitations. Fewer patients were included in the peer coaching arm than the EUC arm, and there was lower overall recruitment rate than intended. This was due to a pause in recruitment for the peer coaching intervention and continued recruitment for the EUC arm (as recommended by our Data and Safety Monitoring Board) during the transition to 2 new coaches. We had planned to make up our recruitment numbers in the peer coaching arm and control for the time of enrollment in the analyses. However, the trial had to be stopped early due to the COVID-19 pandemic, leading to a persistent imbalance between arms and lower recruitment than intended, which decreased the statistical power. Given these power limitations, the secondary and exploratory analyses did not control for multiple comparisons, missing data, or potential confounding. Additionally, weight management program attendance was self-reported.

## Conclusions

This cluster randomized clinical trial of a technology-assisted, low- to moderate-intensity weight management counseling intervention delivered by peer coaches did not result in greater weight loss at 12 months, but it promoted enrollment in a weight management program among an underrepresented, diverse primary care population.

## Notes

Supplement 1.
Trial Protocol

Supplement 2. **eMethods 1.** Participant Inclusion/Exclusion Criteria **eMethods 2.** Recruitment Procedure **eMethods 3.** PAL Intervention Description **eMethods 4.** Clinical and Behavioral Outcome Measures and Procedures **eMethods 5.** Sample Size Calculation **eMethods 6.** Exploratory Analyses **eFigure.** Primary Outcome: Mean Weight Change in kg by Patient Characteristic and Engagement With Intervention **eTable 1.** Baseline Socioeconomic Characteristics and Engagement With Intervention by Race and Ethnicity Group **eTable 2.** Baseline Socioeconomic Characteristics and Engagement With Intervention by Gender Group **eTable 3.** Baseline Socioeconomic Characteristics and Engagement With Intervention by Age Group **eTable 4.** Sensitivity Analyses of Clinical Outcomes at 6 and 12 Months by Treatment Arm (Excluding Patients Using Obesity Medications) **eTable 5.** Sensitivity Analyses of Behavioral Outcomes at 6 and 12 Months by Treatment Arm (Excluding Patients Using Obesity Medications)
eReferences

Supplement 3.
Data Sharing Statement
