# Supplementary material for: Peer Coaching to Support Weight Management in Primary Care: A Cluster Randomized Clinical Trial
Source: JAMA Netw Open. 2025 Sep 2;8(9):e2529136. doi: 10.1001/jamanetworkopen.2025.29136 (PMC12406063; doi:10.1001/jamanetworkopen.2025.29136)
Supplement: Supplement 1. — Trial Protocol [file jamanetwopen-e2529136-s001.pdf]

TITLE: The Peer Assisted Lifestyle (PAL) intervention protocol: A technology-assisted weight-loss intervention within Patient Aligned Care Teams at the VA

(PI: Melanie Jay, MD MS)

SPECIFIC AIMS

Veterans shoulder a disproportionate burden of obesity and its co-morbidities, including diabetes, hypertension, and hyperlipidemia. Modest weight loss in obese patients through diet and exercise improves health and prevents chronic disease, but primary care providers (PCPs) often fail to adequately counsel patients about their weight due to lack of time and training. Thus, tools and brief interventions are needed to support providers' behavior change counseling. The VA currently offers the MOVE! program to treat overweight and obese patients, but fewer than 8% of eligible patients attend. At the same time, Veterans on average see their PCPs 3.6 times per year, which supports the importance of developing primary care (PC)-based interventions. The United States Preventive Services Task force (USPSTF) recommends the use of the 5As framework (Assess, Advise, Agree, Assist, Arrange) for counseling patients about weight.

Interactive behavior change technologies utilizing expert system software programs are an innovative way to facilitate 5As counseling to promote behavior change in primary care. These programs perform computerized risk, lifestyle, and theory- based, behavioral assessment to provide computer-generated, tailored advice to patients. They also can provide information to healthcare teams. The MOVE!11 software is an expert system program for VA patients referred to MOVE!, but is not currently used in primary care by Patient-Aligned Care Teams (PACT).
Collaborative goal setting can be used to achieve behavior change in this intervention. This construct, a critical component of several behavior change theories and models and corresponding to "agree" in the 5As model, has been widely recommended for health promotion in primary care. Our formative work (MIRB #01333) using key informant interviews with PACT teamlets and MOVE! staff and focus groups with Veterans demonstrated that goal setting is feasible and acceptable to patients and PACT teamlets and provided insight on barriers to goal setting, and ways to facilitate goal-setting conversations.

We developed a primary care-based intervention called Peer Assisted Lifestyle (PAL) to facilitate delivery of 5As weight management counseling within primary care and increase adoption of intensive VA programs such as MOVE!. The PAL intervention uses a software tool (that we developed) delivered on tablets to facilitate 5As-based weight management counseling with a Veteran health coach and VA PACT healthcare team to promote goal-setting, behavior change, and weight loss in the primary care setting. The PAL intervention also includes up to 12 health coaching calls to patients over 1 year.

As part of a clustered randomized control trial, we will randomize up to 17 PACT teams to either Enhanced Usual Care or the PAL Intervention, recruiting up to 520 subjects.

STUDY OBJECTIVES

- Test the impact of the PAL intervention on weight change and behavioral/clinical outcomes
- Identify predictors of weight loss in Veterans participating in the intervention group related to goal setting processes and intervention components
- Determine the impact of the PAL intervention on PACT obesity-related counseling practices and attitudes

RESEARCH PLAN

A. BACKGROUND/SIGNIFICANCE

VETERANS/OBESITY

<u>The burden of obesity among Veterans is substantial, and modest weight loss can have significant benefit.</u> The majority of Americans are either overweight or obese[1] and obesity is associated with higher mortality.[2] Approximately 36-37% of patients seen at the VA are obese,[3,4] and obese patients have a high degree of chronic disease. For instance, 84% of obese Veterans have hypertension, 78% have hyperlipidemia, and 45% have diabetes.[4] Modest weight loss (7%)

via a 16-session program can reduce the risk of diabetes in high-risk patients by 58%.[5] Thus, the United States Preventive Services Task Force recommends that all patients are screened for obesity and offered intensive lifestyle counseling.[6]

The VA offers the MOVE! program, an intensive lifestyle behavior change program, nationally. Patients who attended 2 or more MOVE! sessions lost 2.6 pounds more over 6 months than matched controls.[7] They were also more likely to have clinically significant (>5%) weight loss (19% vs. 12%) and less likely to gain weight (29% vs. 38%).[7] Another study of MOVE! demonstrated improved weight loss trajectories (-1.6kg/yr) compared to weight gain (+2kg/yr.) prior to enrollment.[8] Unfortunately, many obese patients are either unwilling or unable to attend intensive weight management programs, and barriers are poorly understood. Only 8% of eligible patients attend at least 1 MOVE! visit[9]. While this is partly due to poor patient adherence to the MOVE! program, another reason is variable implementation of MOVE! across VA sites. In addition, while one of the goals of the MOVE! program was to integrate weight management into primary care (PC), in practice, this program often operates more like a specialty service where treatment is provided outside of the PC visit or setting where patients receive frequent care.

PACT

The PC setting is critical to reducing the burden of obesity; primary care-based interventions have a broad potential reach. The VA/Department of Defense (DOD) guidelines for screening and management of overweight and obesity recommend that providers treat all patients with obesity.[10] Veterans in the VA system see a primary care provider (PCP) an average of 3.6 times per year,[11] providing multiple opportunities for weight management counseling and referral to MOVE!. PC is an important venue to promote weight loss, and effective PC-based interventions can have a significant public health impact. Physicians' and other providers' counseling is associated with positive behavioral and weight-loss outcomes.[12,13] However, PCPs frequently fail to effectively counsel obese patients to lose weight.[14,15] This is due to lack of training,[16] poor competency,[17] perceived lack of effectiveness,[18] and competing demands on time during the medical visit.[19] PCPs may fail to recognize that a patient is obese. In a recent study, only 53.5% of obese patients at the VA had an obesity diagnosis in their electronic medical record.[4] Those that had a diagnosis of obesity were much more likely to receive counseling.[4] Thus, interventions are needed to support physicians' and other providers' obesity identification and counseling.

To improve counseling opportunities and enhance care coordination during a primary care visit, the VA adopted a patient centered medical home model called Patient Aligned Care Teams (PACT) in 2010. The PACT model provides a team-based, patient-centered approach to health care that utilizes a multidisciplinary practice team that includes a RN Care Manager, a Clinical Associate (LPN), and Administrative Associate, and the primary care provider (the "teamlet") as well as social workers, dietitians, and specialists (other team members) to deliver patient-centered care.[9,20] This recent implementation of PACT provides the opportunity for longitudinal, team-based care that is integrated with the MOVE! program. However, in the 2013 MOVE! progress report, the majority (55%) of VA sites surveyed reported that MOVE! programs were separate from PACT.[21] Improving weight management counseling by PACT teamlets could increase patient motivation to attend the MOVE! program and facilitate weight loss for those who do not attend.

THE 5As

The United States Preventive Services Task Force (USPSTF) recommends that providers use the 5As framework to counsel patients for weight management. This model, which has been shown to promote weight loss and smoking cessation,[22,23] guides the provider to Assess risk and stage of change, Advise weight loss and behavior change, Agree on goals, Assist via addressing barriers (motivational interviewing), and Arrange to follow-up or refer patient for further treatment.[24] Our previous work has allowed us to determine which aspects of the 5As framework need to involve the healthcare team (e.g. agree and assist) and also identified which are particularly time-consuming (assessing multiple behaviors and providing advice).

Figure 1: PAL Intervention in the 5As framework

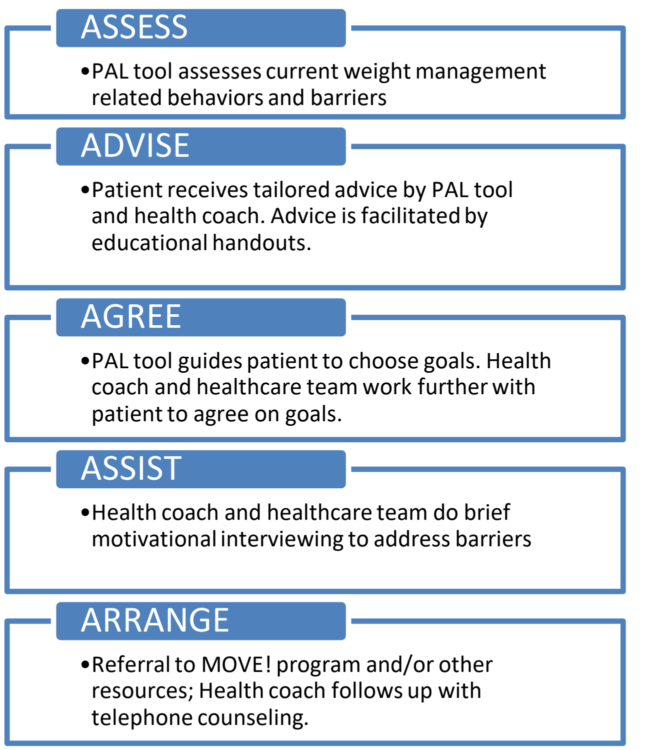

Computerized interventions may be effective in helping deliver 5As-based obesity counseling in the primary care setting. Interactive behavior change technologies use expert system computer software to evaluate patients' risk and current behaviors (assess), and then generate personalized, tailored behavior change advice (advise).[25] They have the potential to help providers counsel obese patients[25] and have been shown to facilitate goal setting (agree) and lifestyle behavior change in primary care settings,[26,27] but have been criticized for not including active provider counseling.[26,27] Patients want and expect their primary care providers to deliver lifestyle and weight-loss counseling[28] and may be more likely to change their lifestyle behavior when counseled by their own provider.[16] Further, patient-provider communication has been directly linked to adherence and health outcomes.[29] Figure 1 shows how the PAL intervention corresponds to the 5As framework.

GOAL-SETTING

Goal setting is a critical component of the PAL intervention , corresponds to the "Agree" component of the 5As framework[24] and is associated with effective weight management.[26,30] Effective weight loss interventions need to include the patients' perspective, and having them set individualized goals is a strategy supported by many behavior change theories including the Theory of Planned Behavior.[31] Based on this theory, having an intention to change a behavior predicts behavior change. Thus, forming specific goals or "goal intentions" increases the likelihood of behavior chage.[32,33] Goal setting is also commonly used to promote behavior change in primary care settings[34] and fits well into other behavior change theories and models including the chronic care model,[35] chronic disease self-management programs,[36,37] and social cognitive theory.[38] A systematic review of goal setting for lifestyle behavior change in primary care showed that it was effective in promoting diet and physical activity changes.[38] Another systematic review showed that technology-assisted interventions combined with counseling promoted weight loss.[39] Thus, technology-assisted goal setting has the potential to overcome barriers and facilitate weight management.
Health-related goals can be general (losing weight, exercising more) or specific (substituting water for soda, attending a weekly aerobics class).[34] Goals can be assigned by a health care provider or set collaboratively. [34] Current goal setting theory, much of which is derived from occupational psychology literature, states that to maximize goal attainment, behavior change goals should be specific, difficult, proximal, and set collaboratively with the provider.[34,37,40,41] However, less is known about how to best apply goal setting to the primary care setting at the VA or the specific socioeconomic,[42] environmental, nutrition knowledge and behavior,[43,44] and health literacy factors[45] that may affect goal setting processes and outcomes. Prior to our own qualitative studies, a review of the literature revealed no studies examining the barriers, preferences, and facilitators to goal-setting in obese VA patients. We used this information to optimize the goal setting process for the intervention.

RELEVANCE OF PROPOSED RESEARCH TO VA

The VA is a national leader in providing comprehensive obesity screening and treatment programs. The VA currently screens all patients for obesity, and high-risk overweight and obese patients are referred by their provider to the MOVE! program to receive comprehensive obesity counseling. Performance measures from the Office of Healthcare Transformation (OHT) stipulate that screening for BMI and offer of referral to MOVE! occur for all eligible patients (the average screening and referral rate is 94%) . The MOVE!11 questionnaire is currently used primarily by the small subset of patients who attend MOVE! programs (use varies by institution). However, this tool is not currently used for PC patients outside of the MOVE! program, and our own formative research showed that it needed increased functionality in order to help patients use the tailored advice to set behavior change goals.

THE PEER ASSISTED LIVING INTERVENTION:

The PAL Intervention will use an online tool to deliver 5As-based obesity counseling. Our systematic review showed that technology-assisted interventions combined with counseling by various members of the healthcare team promoted weight loss. The VA's MOVE!11 software (formerly the MOVE!23) is an example of this technology that has been used as an intake tool for the MOVE! program by thousands of Veterans and utilizes reliable and valid measures to help tailor weight management treatments. Based on the MOVE!11, we created the Goals for Eating and Moving (GEM) tool, optimizing it for use in primary care and adding goal setting functionality to facilitate 5As weight management counseling by PACT teams.

The PAL Intervention will use health coaches integrated within PACT. It is a priority to hire Veteran health coaches from diverse educational backgrounds who will work with individuals to achieve health-related goals. If circumstances restrict availability of a Veteran health coach, we will supplement the intervention with trained, non-Veteran health coaches to maintain frequency of the phone coaching calls. All health coaches can successfully deliver interventions to promote weight management within PC. We will use non-clinician, health coaches integrated within PACT teams to facilitate 5As counseling.

The PAL Intervention will use telephone coaching to facilitate weight management. Higher frequency interventions are associated with better outcomes. Since telephone counseling may be as effective as face-to-face counseling and more cost-effective, the PAL Intervention will incorporate up to 12 telephone coaching calls over 12 months (with initial high frequency) and follow.

B. RESEARCH DESIGN AND METHODS

Figure 3: Study Design Flowchart

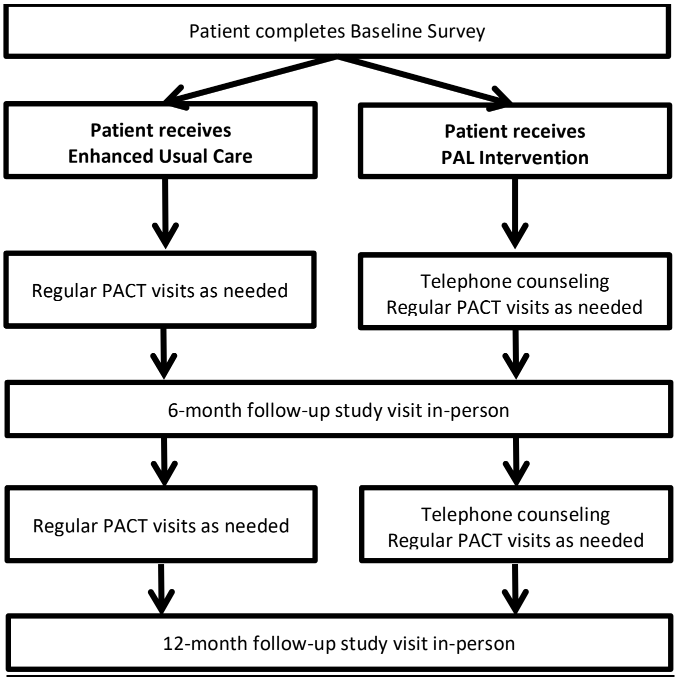

OVERVIEW

<u>Project Objectives</u>

The objectives of this study are to: (a) test the impact of the PAL intervention on weight change and behavioral/clinical outcomes; (b) identify predictors of weight loss in Veterans participating in the intervention group related to goal setting processes and intervention components; and (c) determine the impact of the PAL intervention on PACT obesity-related counseling practices and attitudes.

<u>Project Methods</u>

To achieve these objectives, we will conduct a cluster randomized controlled trial of 17 PACTs (randomized at the provider level) and 520 of their overweight or obese Veteran patients at the Brooklyn campus of the NY Harbor Healthcare System to study the impact of the 12-month PAL intervention when compared to Enhanced Usual Care. Patients in the PAL intervention will complete the GEM tool and receive health coaching, followed by telephone coaching from the health coach over 12 months. The GEM tool is a mobile-friendly software program designed to be delivered on tablet computers in the clinic setting to assess lifestyle behaviors and barriers, provide tailored advice and patient education materials, create initial weight loss and behavior change goals, and facilitate counseling by the health coach and PACT staff. Patients in the Enhanced Usual Care arm will receive patient education materials (standard VA Healthy Living Messages) and information on the MOVE! program. All patient participants will attend follow-up visits at 6- and 12-months to assess Body Mass Index, waist circumference, blood pressure, diet, blood tests, physical activity, and goal attainment. PACT staff and health coach outcomes will include 5As-related competency, as well as quality and frequency of counseling. If circumstances restrict the availability of in-person visits (e.g. COVID-19) or based on Veteran's preference, this study will provide a remote/tele-visit option for the baseline and follow up visits.

DESIGN

Identifying PACT Teams – PACT teams at each site that meet the eligibility criteria below will be selected to be enrolled in the study. Individual PCPs will be allowed to opt out of the study. We do not anticipate that primary care providers will opt out or drop out once enrolled based on the VA PROVE study where all 18 eligible RN care managers and 51/52 PCPs participated for the duration of the study.[10]

Exclusion criteria

- PCP does not agree to participate.
- Not seeing a general primary care population with at least 20% of Veterans eligible for the study (e.g., will exclude Geriatrics, MH, ID, and HBPC)
- PCPs with less than 250 patients

Identifying and Recruiting Veterans – Using the Veterans Health Information Systems and Technology Architecture (VISTA), the VA's Health Information Technology system, we will continuously identify Veterans who meet the eligibility criteria below. Research staff will review charts to confirm eligibility and send lists to their PCPs who will identify other contraindications to participating. One to two weeks before their potential baseline visit, potential participants will be sent a letter signed by the Principal Investigator (PI) describing the study and giving them the opportunity to opt out (i.e. request not to be contacted). We will then call Veterans to recruit, screen for eligibility, and schedule the baseline visit which allows enough time for all intervention components to be completed. A similar recruitment strategy was used in the VA PROVE study,[10] as well as in our focus group recruitment. We have IRB approval to use this strategy, and successfully recruited Veterans using this protocol in our pilot studies. We will aim for 20% of our sample to be comprised of women so that we can explore whether the intervention needs to be tailored differently for women.[46] To oversample women, we will contact a random sample of eligible male Veterans and all eligible female Veterans consecutively (in a 5:1 ratio). With consultation from local and national leaders in women's health research, we will provide tailored recruitment materials, monitor recruitment targets, and make iterative changes to our recruitment strategy if these targets are not met.

Inclusion Criteria

- Age 18-69 (this age range represents MOVE! eligibility)[43]
- BMI of ≥30kg/m^2 or

  OR
- BMI of ≥25kg/m^2 AND one of the following obesity-associated conditions: [10,58]
  - Hypertension: 401-405
  - High Cholesterol: 272
  - Sleep Apnea: 780.57
  - Osteoarthritis: 715.9
  - Metabolic Syndrome: 277.7
- Under the care of PCP with at least 1 prior visit with the provider in the past 24 months
- Access to a telephone
- Be patients of the New York Harbor Brooklyn campus only

Exclusion Criteria (note that we are no longer excluding Veterans with specific health issues)

- Non-Veterans
- A documented current history of active psychosis or other cognitive issues via ICD-10 codes
- Has participated in ≥4 MOVE! sessions in the past year
- Pregnancy

- PCP stating that Veteran should not participate
- Self-reported inability to read at a 5th grade level due to literacy level or vision problems

With an expected 20% participation rate (based on pilot data) and a 75% retention rate (based on rates from another VA study and other technology-based interventions) [39,41], we will need to screen 2,615 Veterans with overweight/obesity to enroll a baseline sample of up to 520 Veterans and end with 392 Veterans completing the study. With a screening rate of approximately 30-35 Veterans per week (6 women, 24 men), we will be able to recruit our target population by the middle of Year 3 (recruitment will start the first quarter of Year 2).

Study Arms/Randomization – Under the direction of the study statistician, the PACT teams will be randomized at the provider level using a random number generator to either the PAL Intervention or Enhanced Usual Care intervention arms.

- *PAL Intervention group*: Participants will arrive and complete consenting materials, a Baseline Survey, and the GEM tool. They will then have a brief break if needed. After the break, they will work collaboratively with the Health Coach using tool-generated materials to modify their goals into SMART goals (specific, measurable, attainable relevant, and timely). The patient will then receive materials containing an individualized report with a summary of their personalized advice, initial goals, and barriers and facilitators to weight loss. They will also receive a packet of MOVE! handouts tailored to their questionnaire responses and schedule 10-12 follow-up health coaching phone calls during the next 12 months of intervention. The Health Coach will also inform the patient of the resources available to them at the VA (e.g. MOVE, TeleMOVE!) that can assist them in achieving their goals.

  During the baseline visit we will encourage patients to sign-up for MyHealth*e*Vet, if they have not already done so. If patients are signed-up for MyHealth*e*Vet, they can choose to receive reminders for their upcoming study visits or health coach phone calls electronically via myHealth*e*Vet. Alternatively, patients can choose to receive reminder phone calls from secure VA phones lines. Before upcoming phone coaching calls and study visits, we will send reminders via the secure messaging system MyHealth*e*Vet or make phone calls via secure VA phone lines.

  At the end of the visit, the Health Coach will enter a research note into the CPRS system detailing the patient's goals and other important information in the form of a provider report for the provider to review (generated by the tool). At a later date, the patient will meet with a PACT member (either the PCP or another participating PACT member) during their regularly scheduled PC visit who will review the goals and agree on them and/or modify their goals based on a collaborative discussion and conduct brief motivational interviewing to address potential barriers (3-5 minutes). The PACT member will summarize this interaction in a specific CPRS note. Also, over the next 12 months of the intervention, the PACT teamlet will receive CPRS reminders to discuss the goals with the patients when they return for their regularly scheduled PC visits. Patients will follow up with VA resources if indicated/desired. PAL intervention participants will return for separate 6 and 12 month follow-up study visits to measure intermediate behavior change and weight outcomes.

  If circumstances restrict the availability of in-person visits (e.g. COVID-19) or based on Veteran's preference, this study will provide a remote/tele-visit option for the baseline, 6 and 12 month follow up visits. For the remote baseline visit, a scale, a tape measure for the waist circumference, informed consent form documents, and the study resource materials will be mailed to the participant. Participants who enroll via a remote visit will have IRB approval for written consent documentation to be waived anda verbal assent will be obtained through telephone/video confirmation by reading the informed consent form to the participant. Through the verbal assent process, the study will document in the patient's medical records on the willingness to be contacted for future research about similar weight loss management research.

- *Enhanced Usual Care*: Participants will complete the same consenting materials and Baseline Survey as the intervention group. Instead of the completing the PAL tool and receiving health coaching, they will receive

weight management handouts (Healthy Living Messages handouts and MOVE! handouts) as well as information about intensive weight management programs at the VA including MOVE! Participants will follow-up with their PACT teamlets as needed when they return for their regularly scheduled PC visits. Patients will follow up with VA resources if indicated/desired. Enhanced Usual Care participants will return for separate 6 and 12 month follow-up study visits to measure intermediate behavior change, and weight outcomes.

If circumstances restrict the availability of in-person visits (e.g. COVID-19) or based on Veteran's preference, this study will provide a remote/tele-visit option for the baseline, 6 and 12 month follow up visits. For the remote baseline visit, a scale, a tape measure for the waist circumference, the informed consent forms, and the resource materials will be mailed to the participant. Participants who enroll via a remote visit will have IRB approval for consent documentation to be waived and verbal assent will be obtained through telephone/video confirmation by reading the informed consent form to the participant. Through the verbal assent process, the study will document in the patient's medical records on the willingness to be contacted for future research about similar weight loss management research.

PAL INTERVENTION ARM: BASELINE VISIT

For Veterans in the PAL Intervention, they will need to complete the following (items 1 and 2 will be completed at the baseline visit). Average times to complete are included below based on pilot testing.

1. Complete the PAL tool (20 minutes)
2. Meet with a health coach and review materials with tailored educational materials (30 minutes)
3. Receive 10-12 telephone coaching calls from health coach (20-30 minutes each)
4. Follow up as needed with PACT teams for PC (varies)

Baseline Health Coach Visit – After completing the PAL tool, a health coach meets with the Veteran for approximately 30 minutes (based on our pilot study). All sessions will be audio-recorded for fidelity checks and health coach training if recording equipment is available. The health coaching manual will help the coaches to do the following tasks:

- Make initial goals into SMART goals (Specific, Measurable, Attainable, Relevant, Timely)[47] using worksheets
- Encourage participation (using MI) in MOVE, TeleMOVE!, or other VA or community-based programs/gyms
- Teach how to self-monitor weight, diet, and PA behaviors via pedometer, food log, and/or weight management apps (e.g., MOVE! Coach[48,49] on iPhones, My Fitness Pal[50] on Androids)
- Provide brief MI and Brief Action Planning[51] to address barriers and increase Veteran self-efficacy
- Facilitate meetings with PACT dietitian if needed
- Enter a report for PACT teams into CPRS summarizing the encounter (generated by the PAL tool)
- Communicate potential barriers/concerns to the PACT team
- Encourage utilization of MyHealth*e*Vet
- After baseline visit, provide 10- 12 telephone coaching calls (described below)

Telephone Coaching/Self-Monitoring Visit – To achieve sufficient intensity according to VA/DOD and USPSTF guidelines,[19,26] we will incorporate 12 telephone coaching calls by a health coach over 12 months. Scheduled calls will occur two weeks and four weeks post-baseline, then once every month for the remainder of the study. PAL Intervention arm Veterans will receive a reminder call to self-monitor their weight, food intake, and PA for at least 3 days prior to the coaching call. Studies suggest that episodes of short, consistent self-monitoring (for 3 days) lead to weight loss and may promote better adherence.[63] Health coaches will use self-reported self-monitoring data to determine goal adherence and counsel Veterans to encourage small changes.[39] Data from health coaching sessions will be documented in REDCap, our research database. They will help Veterans create new goals when appropriate and use MI techniques to address barriers to behavior change. Veterans will be able to contact health coaches for additional support.

CPRS Research Note/Reminders– The PAL tool creates a provider report that the health coach will cut and paste into CPRS as a research note to summarize and communicate information to the Veteran's PACT team about the health coach session. This note generates a CPRS reminder for the PCP. The reminder facilitates documentation about whether the PACT team discussed goals and provided further counseling. This research note and reminder system will also be used to document both health coach telephone counseling and PACT counseling during future PC visits.

PACT Team Role – PACT teams randomized to the intervention arm will provide at least 3-5 minutes of counseling. They will choose who on the team counsels (based on formative data indicating variability in team member roles). They will document counseling with the help of CPRS reminders, provide brief MI to address barriers (depending on time and Veteran needs), and briefly discuss weight management goals during future PC visits. Each PACT team dietitian will co-sign the health coach CPRS note and will be available as a resource. Health coaches will attend PACT meetings to communicate the progress of enrolled Veterans. They will also communicate PACT team barriers to the PI.

Tailoring the PAL Intervention for Participants with Diabetes– We will use data from Veterans with diabetes from prior pilot studies (unpublished data) and input from our Executive Committee to develop additional diabetes-related content. We will also revise the health coach curriculum to include common counseling needs and concerns for patients with diabetes and linkages to diabetes resources at the VA. Gail Schechter, RD, the MOVE! coordinator, and Joanna Dognin, PhD, a behavioral psychologist and Health Behavior Coordinator (HBC) at the VA NYHHS, both Co-Is, will help create the content and curriculum.

PACT Team Training – PACT team staff will receive training sessions during Year 1 of the study with follow up training by Drs. Melanie Jay (PI) and Joanna Dognin (Co-I). As part of her role as HBC, Dr. Dognin has provided the majority of RN care managers and PCPs 2-4 hours of MI training, as well as 6 hours of TEACH for Success Health Coaching Course[64] during the past 4 years. We will build upon this VA training to include 5As-based obesity counseling and additional MI skills practice based on a 5As training intervention conducted by Dr. Jay[36] and other adapted MI materials through CCMI.[52] We will invite PACT staff to observe MOVE! sessions since we and others have found that clinicians often lack knowledge about program specifics which negatively impacts referrals.[8,66] Training will also include indications and processes for referring to bariatric surgery. The first training will be approximately one hour with 2-3 subsequent trainings for 15-30 minutes. We will provide an overview of the 5As and the intervention components and practice brief MI (using role playing) to support Veterans' weight management goals and address barriers to change. Finally, we will assist in incorporating the health coach into each PACT team (e.g., include health coach at monthly PACT team meetings). We will use an academic detailing approach scheduled either one-on-one or in small groups so as not to interfere with VA clinical or administrative duties. This delivery approach is well accepted and similar to that used in the PROVE study.[10]

Data Collection

Veterans: Assessment will occur at the following time points:

- In person study visits at Baseline, 6, and 12 months: Veterans will meet with research staff to complete surveys (described below), weight, height (Baseline only), blood pressure and waist circumference measurement, and blood tests (Baseline, 6, and 12 months only). If circumstances restrict in-person visits (e.g. COVID or if Veteran opts for a remote visit, the weight and waist circumference will be obtained via telephone/video. Height and blood pressure will be obtained through the patient's most recent VA visit from the electronic medical record. The Baseline will include a survey (completed before receiving PAL Intervention/Enhanced Usual Care). Veterans will receive accelerometers (described below). Electronic chart review will also occur at these time points. At 6 months, Veterans in the intervention arm will participate in a short qualitative interview as part of the RE-AIM evaluation Telephone Coaching Calls (Veterans in PAL Intervention arm only): Health coaches will use a counseling tool that also facilitates data collection.
  Chart reviews (24 months post-baseline visit) to explore maintenance of weight and focus groups post-

intervention

PACT Teams: At 6 and 12 months, we will give surveys to PCPs and audiotape select visits.

Measures

All variables described below will be collected and analyzed by research staff. Core measures are listed and described below. Baseline surveys have been pilot tested in 45 Veterans.

BASELINE DEMOGRAPHIC DATA – We will collect socio-demographic information, health literacy (2-item screen[53]), and depression (PHQ970) via surveys. Co-morbidities will be determined via electronic chart review.

AIM 1: WEIGHT AND CLINICAL OUTCOMES –

- Weight & Height: RAs (in-person visit) will obtain Veteran weight measurements using a standardized protocol which includes: 1) weighing without shoes or heavy garments using a digital scale that will be calibrated monthly; 2) at each study time point (Baseline, 6, and 12 months). Height will be measured with a stadiometer and rounded up to the nearest half-centimeter, at Baseline only.
- RAs (remote visit) will obtain Veteran weight measurements via telephone/video using a standardized protocol which includes: 1) weighing without shoes or heavy garments using a digital scale; 2) at each study time point (Baseline, 6, and 12 months). Height will be obtained through chart review. Waist Circumference: Waist circumference will be measured using procedures adapted from National Health and Nutrition Examination Survey (NHANES).[54] RAs (in-person visit) will measure to the nearest quarter inch at the high point of the iliac crest at minimal respiration. This will be measured at Baseline, 6, and 12 months. RAs (remote visit) will guide the Veteran on how to take the waist circumference measurement and obtain measurements through telephone/video.

- Blood Pressure: RAs (in-person visit) will obtain 2 blood pressure measurements using an automated sphygmomanometer (Omron HEM-907XL). The cuff will be placed on the Veteran's left upper arm, with the bottom of the cuff placed approximately 1 in above the crook of the elbow as per a standard protocol.[55]
RAs (remote visit): Blood pressure will be obtained through chart review from the Veteran's most recent visit to the VA.

COMPENSATION

To compensate for travel and time spent completing survey measures and basic measurements, study participants will be given a cash voucher in the amount of 60 dollars for the Baseline study visit, 45 dollars for the 6 month study visit, and 50 dollars for the 12 month study visit. This proposed payment is reasonable and commensurate with the expected contributions of participants and is meant to provide additional incentives for participants to complete all 3 study visits. This amount of payment and the terms of the payment are included in the informed consent form. This payment is fair and appropriate and does not constitute undue pressure or influence, or coercion of, the prospective research participant to volunteer for or continue participation in the research study.

POTENTIAL BENEFIT/RISK

Benefits:

- *Patients*: The patients who participate in the study will have the opportunity to receive weight management information, and potentially set lifestyle behavior change goals to improve their diet and increase physical activity, which may lead to weight loss and improved health outcomes. Even for patients who do not change their lifestyle behaviors, talking about these topics with trained researchers could serve as support or motivation to move them closer to doing so in the future.

- *Providers and other VA employees*: VA employees who participate in the study may gain improved obesity-related knowledge and patient counseling skills, which could enhance their career, job performance, and satisfaction. Specifically, VA employees may have the opportunity to receive (additional) training in 5As weight management counseling and practice brief motivational interviewing. Training in these could help to facilitate

and/or improve discussions around weight management and health behavior change with patients, as well as encourage the use of individualized techniques to improve diet and exercise and setting health behavior change goals with patients.

Risk:

- *Patients:* This research involves minimal risk for physical, psychological, social, and economic harm. The researchers understand that exploration of these topics and a persons' individual struggle with their weight can be emotionally charged for many people, particularly considering the stigma placed on obesity in our society. The researchers have been trained in order to effectively facilitate conversations on this sensitive topic and will seek to minimize any emotional discomfort you may feel during the study. Additionally, any potential behavior changes related to diet or exercise will be assessed and approved by properly trained individuals including select research staff, the Primary Investigator, and health professionals. Patients will be encouraged to slowly increase their physical activity under the supervision of their PCP. While there is always the risk of injury from starting or increasing physical activity, the benefits usually outweigh potential harms. We will monitor patients for adverse events.

- *VA employees*: This research involves minimal risk for physical, psychological, social, and economic harm.

MEASUREMENTS

Assessment will occur at the following time points:

A. Baseline study visit:
   a. In person: baseline survey, basic physical measurements (weight, height, waist circumference, and blood pressure).
   b. Remote visit: baseline survey, basic physical measurements (weight and waste circumference) via telephone/video, height and blood pressure results through chart review
B. Follow-up study visits at 6 and 12 months:
   a. In person visit: Follow-up in-person questionnaire and basic physical measurements (weight, waist circumference, blood pressure).
   b. Remote visit: Follow-up questionnaire, basic physical measurements (weight and waist circumference) via telephone/video, blood pressure results through chart review
C. Follow-up telephone calls (only patients in PAL intervention): Follow-up telephone questionnaire
D. Chart review: This will occur periodically to assess MOVE! attendance, PACT teamlet visits (documentation of weight management-related conversations), and medical co-morbidities. Outcomes will be assessed through chart review up to 2 years after the baseline visit.

Baseline Data: Prior to the intervention, patients will receive a Baseline survey. To evaluate recruitment for the intervention, baseline data including socio-demographic information, perceived health status, health literacy, perceived environmental factors, and depression will be collected.[56-59] Co-morbidities will be determined through chart review of the electronic medical record. We will also collect health-related attitudes, beliefs, and baseline behaviors.

Weight & Height: In person visit, study personnel will obtain patient weight measurements using a standardized protocol which includes; 1) weighing without shoes or heavy garments using a digital scale that will be calibrated monthly; at each study time point (Baseline, 6, and 12 months). 2) Height will be measured using the primary care clinic stadiometer and rounded up to the nearest half-centimeter at Baseline. For remote visits, study personnel will obtain Veteran weight measurements via telephone/video using a standardized protocol which includes: 1) weighing without shoes or heavy garments using a digital scale; 2) at each study time point (Baseline, 6, and 12 months). Height will be obtained through chart review.

Waist Circumference: Waist circumference will be measured using procedures adapted from National Health and Nutrition Examination Survey (NHANES)[54]. In person visit, study personnel will use measuring tape to the nearest quarter inch at the high point of the iliac crest at minimal respiration. This will be measured at baseline, 6, and 12 months. For remote visits, study personnel will guide the Veteran on how to take the waist circumference via telephone/video.

Blood Pressure: Study personnel will obtain 2 blood pressure measurements using an automated sphygmomanometer (Omron HEM-907XL). The cuff will be placed on the Veteran's left upper arm, with the bottom of the cuff placed approximately 1‖ above the crook of the elbow as per a standard protocol.[55] For remote visits, study personnel will obtain blood pressure through chart review from the Veteran's most recent visit to the VA.

Feasibility: We will use process measures from direct observation to determine the feasibility of implementing the PAL intervention at the initial visit. Data on the time to complete the PAL tool, number and types of goals made, number of patients reporting that their PACT teamlets engaged in goal setting conversations, number of reminders completed by the PACT teamlets, time spent doing the intervention, the impact of the intervention on workflow (physician report, qualitative interviews) will be collected. At 6 months into the study period, participating PACT teamlet staff will complete an attitudes survey based on one we have used previously pertaining to intervention implementation. We will also perform qualitative interviews with providers. We will use chart review to note the number of times patients in the telephone support arm actually receive telephone counseling.

Acceptability: We will collect data on patient satisfaction with the intervention and the overall visit[60,61] using patient and provider surveys. We will conduct interviews with participating providers to obtain qualitative data on acceptability.

Intermediate Outcomes: Our intermediate outcomes are based on the theory of planned behavior [31] where motivation and intention to perform a behavior, mediated by self- efficacy, predict behavior change. These constructs will be measured with previously used items that have been adapted for our survey measures by adding other elements of the Theory of Planned Behavior including behavioral beliefs, attitudes, subjective norms, and perceived behavioral control. We will also assess goal attainment at 6 and 12 months.

Physical Activity (PA) Outcomes: We will measure duration and intensity using the International Physical Activity Questionnaire (IPAQ) items[62] and accelerometers[63]. The ActiGraph Link (GT9X) accelerometer, worn on the wrist, will be used to objectively measure PA for 7 days at Baseline, and at 6 and 12 months. Study staff will place the Link monitor on the non-dominant wrist to measure PA for 24 hours a day during the Baseline 7-day measurement period. Veterans will then mail the monitor back to the study team in a supplied mailer with postage. At 6 and 12 months, the accelerometer will be distributed to the Veteran with a supplied mailer with postage to be mailed back to the study team. Veterans will not be able to see their data on these monitors so as to minimize measurement influence on PA behaviors. This approach has been used in the NHANES Physical Activity Monitor study.[54] The acceleration data will be used to characterize PA behavior. Examples of PA behavior include steps per day, ambulatory time, as well as daily sum of vector magnitude units per day. The daily sum of vector magnitude units are a quantification of all movement including ambulatory and non-ambulatory activity.

Behavioral Outcomes: We will assess diet and physical activity behaviors at Baseline and again at 6 and 12 months. Our main dietary outcomes will be servings of fruits and vegetables, fat intake, refined carbohydrates, and sweetened beverage intake via surveys. A subset of up to 20 participants will complete 24-hour dietary recalls, and this will allow us to assess total energy intake and energy density in this subgroup. Physical activity outcomes will be changes in duration and intensity. We will use chart review to evaluate attendance to TeleMOVE! or MOVE! (# of sessions) pre- and post-baseline visit and 6 and 12 months post- intervention.

Treatment Fidelity: We will do detailed chart reviews using a chart abstraction tool to monitor how often providers see patients and document goal-setting discussions during subsequent visits after the index visit.

Data Storage/Security

- *Research Activities:* The following will all take place in private room at the Brooklyn VA with the PI and/or research staff – filling in consent forms, using PAL tool, receiving weight management information, receiving weight management counseling, completing survey measures.

- *Storage of data:* All written data will be kept in locked filing cabinets in a locked room only accessible by the PI and approved study personnel and all electronic data (including survey responses, audio files, and responses to online tool) on secure local VA servers and only accessible on VA password protected computers by the PI and study personnel. Original audio files will be removed from recorders. The data key link will be maintained as a password-protected Excel datasheet on a secure VA Harbor computer within a locked VA Harbor office by the PI, and destroyed upon completion of the final study dataset. The data key link will be kept separate from all survey responses. Only study investigators will perform data entry, access either the surveys or datasets, or perform any analysis of the dataset.

- *Transcription:* For transcription of audio files, files will be sent as encrypted files through the secure server to the VA-contracted transcription company, Transcription Outsourcing, LLC. Transcripts will be de-identified by leaving all identifiable information out of the transcript and using only a unique coded identifier generated by the Principal Investigator. This unique identifier will not use any identifying information (i.e. it will not be generated using the subject's social security number, name, etc.) Recorded information will be transferred to Transcription Outsourcing via a HIPAA-compliant web portal using a VA computer. The transcripts will then be stored and analyzed on a VA secure server. This transcription procedure was approved by the IRB previously (MIRB 01333) under the consultation of the ISO and PO.

- *The PAL tool*: The PAL tool uses a web-interface to ask health questions and collect the data in order to deliver tailored advice. This website will be hosted on either a hired private programmer's server (approved research personnel) or an NYU server. In both cases, Data Use Agreements have been setup with both parties and the VA to protect ownership and use of collected coded data. Data will be regularly migrated from either server (private programmer or NYU) to the VA server via encrypted USB drive. This data storage procedure was approved by the IRB previously (MIRB 01333) under the consultation of the ISO and PO.

Other Quality Control Procedures: The study staff will meet biweekly for the duration of the study to review patient recruitment and data collection procedures to ensure standardization. Research assistants will send reminders to Health Coaches when it is time to call patients in the telephone support arm. To monitor for and address unanticipated adverse events, there will be discussions during meetings with research staff, PI, mentors of PI, and providers/VA staff.

DATA ANALYSIS

Sample Size and Power Analysis: We base our sample size on within-person weight change at Baseline and 12 months in each arm. We assume an interclass correlation coefficient (ICC) of 0.05 for Veterans within each PCP and an ICC of 0.01 for PCPs within each PACT team. Assuming that at the end of the study we have 5 teams in each intervention arm and recruit and obtain baseline data for 52 Veterans per PACT team (392 Veterans total at 12 months), we will have sufficient power to detect a 2.2kg (SD=6.0kg) difference in weight between the intervention and non-intervention arms. If we do not have PACT attrition (5 PACT teams per arm), then we will have additional power. This amount of weight loss is consistent with findings from the ASPIRE VA study[39] and a systematic review of technology-assisted weight loss interventions in primary care.[41] We will also have the power to detect our hypothesized difference of 15% of PAL Intervention Veterans achieving 5% weight loss (clinically significant—as little as 5% weight loss can improve cardiovascular risk), 85% assuming 12% of intervention arm Veterans achieve this amount of weight loss.[23] We will be able to detect 2.9cm change in waist circumference.

Statistics Analysis: First, all the variables will be summarized using mean with standard deviation and median with interquartile range for continuous variables and frequency table for categorical variables, overall and by randomized arms, respectively. Mann-Whitney tests for continuous variables and Fisher exact tests for categorical variables will be used to check if baseline characteristics are balanced between two arms. Second, Mann-Whitney tests for continuous outcomes and Fisher exact tests for categorical outcomes will be used to compare outcome variables (including intermediate outcomes, behavioral outcomes, and weight outcomes) between two arms at 6 months and 12 months, respectively. Wilcoxon rank tests for continuous outcomes and McNemar tests for binary outcomes will be used to compare them between follow-ups and baseline within each arm. Third, repeated measures modeling based on mixed models will be conducted to compare outcome variables between two arms, utilizing all the data at baseline and three follow-ups. Such modeling can adjust for baseline characteristics, take into account the correlation among repeated measures within subjects, and deal with missing data automatically assuming missing at random. Finally, we will conduct sensitivity analysis based on multiple imputation procedures to examine the impacts of missing data under some missing not at random assumptions.

We will conduct several statistical analyses to describe Veterans participating in the study. Descriptive statistics, including numerical summaries, frequency tables and graphical displays, will be used to present baseline participant characteristics. Exploratory tests, such as chi-square tests and t-tests, will be used to investigate if baseline characteristics are balanced between two randomized arms. Analysis of missing data due to dropout or incomplete surveys will be investigated by inferring a relationship between baseline characteristics/risk factors and 0-1 indicator that defines whether or not a measurement is observed. This analysis will help to identify factors that are likely to be associated with dropout. Additionally, we will investigate whether the dropout rates are different between two arms. For instance, participants with higher levels of motivation may be more likely to be retained in the study. To deal with these missing, we will incorporate the multiple imputation procedure with the regression analyses to be discussed in the following, along with some sensitivity analysis.

In order to assess the impact of neighborhood-level covariates that may help predict weight loss and behavior change, as well as to better understand and report the environmental context of our study participants, we will determine the Veteran's census tracts and block groups. To do this, we will send a csv file containing the patients' addresses to the VA's geocoding service, Geospatial Service Support Center (GSSC), which uses VA's secured proprietary Enterprise Geographic Information System (GIS) software (https://www.va.gov/oei/docs/va-spatial-data-strategy.pdf), via the VA's encrypted email. The data will not include the patient's name or apartment number. GSSC will process the data and return a copy of the spreadsheet with the census tract and block group codes added. Once the data is geocoded, the addresses will be purged from the GSSC's database. All applications of geospatial data will be compliant with the objectives and standards established under the VA Spatial Data Strategy (2022).

Qualitative Data: The audio recordings of provider interviews and patients visits will be transcribed by a VA-contracted transcription company (methods used in our previous IRB-approved study MIRB 01333 and MIRB 01496). We will use detailed grounded theory-based procedures including open, axial, and selective coding.[61] After multiple readings of the transcripts and making marginal notes with a second researcher, we will develop, test, and refine a systematic coding scheme of themes and sub-themes, and relationships thereof using NVIVO qualitative data analysis software accessed through VINCI to assist in data analysis and interpretation. Analysis will be facilitated through constant comparison techniques.

EXPLORATORY AIM

Exploratory Aim**:** To compare the intervention arm to a third study arm of non-enrolled PC patients matched for age, gender, and BMI ("Usual Care").

Analyzing the Third Arm: We will compare the two randomized study arms (Enhanced Usual Care and PAL intervention) arms with the third study arm of non-enrolled PC patients who are from the same cohort (seen by the same providers and eligible according to the same inclusion and exclusion criteria). Because the third study arm is not randomized, we do not expect the three arms are balanced at baseline characteristics and comparable. We will first identify important

confounders (such as age, gender, BMI) and then use propensity score to adjust for these confounders when comparing the outcomes between the three arms.

STATISTICAL AND DATA ANALYSIS

First, all the variables will be summarized (intention-to-treat approach) using mean (with standard deviation) and median (with interquartile range for continuous variables and frequency table for categorical variables) overall and by interventions arms, respectively. Then, Mann-Whitney tests for continuous variables and Fisher's exact tests for categorical variables will be used to check if both providers' and Veterans' baseline characteristics are balanced between the intervention arms.

Specific Aims Analysis (main outcomes will be analyzed using intention-to-treat methodology).

1. Test the impact of the PAL intervention on weight change, clinical, and behavioral outcomes

1a: Veterans in the PAL Intervention arm will lose 2.2 kg and 27% will achieve ≥5% weight loss after 12 months of treatment. There will be 0kg weight change in the Enhanced Usual Care arm, and 12% will achieve ≥5% weight loss.

1b: The PAL Intervention will result in improvements in behavioral outcomes (e.g., increased number of steps, increased fruit/vegetable intake, increased attendance to MOVE!) and clinical outcomes (blood pressure, Low Density Lipoprotein (LDL), Hemoglobin A1C, and waist circumference).

1c: The PAL Intervention will increase attendance to MOVE! and/or other intensive programs.

Method: The primary outcome is mean weight loss at 12 months. Mann-Whitney tests for continuous outcomes (e.g., weight loss) and Fisher's exact tests for categorical outcomes (e.g., whether or not Veterans achieve ≥5% weight loss) will be used to compare the two intervention arms at 12 months, respectively. Unadjusted confidence intervals will be computed to measure the effects of the PAL intervention on the outcomes compared with the Enhanced Usual Care. Repeated measures modeling based on mixed models will be conducted to compare outcome variables between intervention arms, utilizing all of the data at Baseline and at follow-up visits. Such modeling can adjust for baseline characteristics (e.g., diabetes, gender), taking into account: a) the correlation among Veterans within providers, b) the correlation among repeated measures within Veterans. Model-based adjusted confidence intervals will be provided to measure the effects of the intervention on the outcomes as well. Although repeated measures modeling can address missing data automatically, assuming it is missing at random, we will use a multiple imputation procedure to conduct sensitivity analyses under the practical assumption of missing not at random. The sensitivity analyses will be conducted using pattern-mixture models to examine if the statistical findings are robust when considering several scenarios, including the least-favorable scenario where the missing data from the PAL intervention arm were to follow the same pattern as that of the observed data from the Enhanced Usual Care arm.

2. Identify predictors of weight loss in Veterans participating in the intervention arm related to intervention components and goal-setting processes

2a: Participation in MOVE!, number of telephone coaching calls received, the use of self-monitoring, and counseling by PACT teams will be associated with weight loss.

2b: Veteran self-efficacy and goal attainment will be associated with weight loss.

Method: Visualization tools such as scatterplots and descriptive analyses such as Spearman correlation coefficients will be used to display associations between weight change and potential predictors. Multivariate regression models will be used to examine the effects of those predictors that are suspected to be associated with weight loss in the intervention arm. Multivariate linear regression models will be considered for continuous outcomes and multiple logistic regression models will be considered for binary outcomes. We will construct classification and regression trees (CARTs), which will generate the variable importance for each intervention component. Missing data will be dealt with using inverse-probability-weighted methods.

3. Determine the impact of the PAL Intervention on provider and nurse obesity-related counseling practices and attitudes

3a: Veterans in the PAL intervention arm will receive more frequent and higher quality weight management counseling from PCPs than those in the control arm. The intervention will improve PCP self-efficacy, attitudes, and perceived competency in weight management counseling.

Method: Mann-Whitney tests for continuous provider-level outcomes and Fisher's exact tests for categorical provider-level outcomes will be used to compare the two intervention arms at each survey. Confidence intervals of the effects will be computed as well.

Missing Data Analysis (to adjust for response bias) -- Although the repeated measures modeling can address missing data automatically under the assumption of missing-at-random, we will further analyze the missing data due to loss of follow-up or non-response using two popular missing data tools, inverse-probability-weighting approach and multiple-imputations approach. Both missing data approaches utilize the baseline characteristics to adjust for response bias. In addition, we will use multiple-imputations procedure to conduct sensitivity analyses under the practical assumption of missing not at random. Specifically, the sensitivity analyses will be conducted using pattern-mixture models to examine if statistical findings are robust when considering several scenarios, including the least-favorable scenario where missing data from the intervention arm were to follow the same pattern as that of the observed data from the Enhanced Usual Care arm.

Sample Size and Power Analysis – We base our sample size on within-person weight change at Baseline and 12 months in each arm. We assume an interclass correlation coefficient (ICC) of 0.05 for Veterans within each PCP and an ICC of 0.01 for PCPs within each PACT team. Assuming that at the end of the study we have 5 teams in each intervention arm and recruit and obtain baseline data for 52 Veterans per PACT team (392 Veterans total at 12 months), we will have sufficient power to detect a 2.2kg (SD=6.0kg) difference in weight between the intervention and non-intervention arms. If we do not have PACT attrition (5 PACT teams per arm), then we will have additional power. This amount of weight loss is consistent with findings from the ASPIRE VA study[39] and a systematic review of technology-assisted weight loss interventions in primary care.[41] We will also have the power to detect our hypothesized difference of 15% of PAL Intervention Veterans achieving 5% weight loss (clinically significant—as little as 5% weight loss can improve cardiovascular risk), 85% assuming 12% of intervention arm Veterans achieve this amount of weight loss.[23] We will be able to detect 2.9cm change in waist circumference.

If we assume the missing rate at 12 months is 15%, then we will have 44 evaluable Veterans per team. We assume an interclass correlation coefficient (ICC) of 0.03-0.04 for Veterans within each PACT team and an ICC of 0.01 for PCPs within each team. If the ICC of Veterans within each PACT team is 0.03, the power is 85% to detect a 2.2kg (SD=6.0kg) difference in weight between the intervention and intervention arms; the power is 87% to detect a 2.9cm (SD=7.7cm) difference in waist circumference. This amount of weight loss and waist circumference change is consistent with findings from the ASPIRE VA study and a systematic review of technology-assisted weight loss interventions in primary care.5 In addition, the power is 91% to detect our hypothesized difference between 25% Veterans in the intervention arm and 10% of Veterans in the control arm achieving 5% weight loss (clinically significant). If the ICC of Veterans within each PACT is 0.04, the powers are 81%, 83%, and 87% to detect the same effect sizes of weight loss, waist circumference, and proportion of 5% weight loss, respectively.

REFERENCES

1. Das SR, Kinsinger LS, Yancy WS, et al. Obesity prevalence among veterans at Veterans Affairs medical facilities. Am J Prev Med. 2005;28(3):291-294.

2. Noël PH, Copeland LA, Pugh MJ, et al. Obesity diagnosis and care practices in the Veterans Health Administration. J Gen Intern Med. 2010;25(6):510-516.

3. Knowler WC, Barrett-Connor E, Fowler SE, et al. Reduction in the Incidence of Type 2 Diabetes with Lifestyle Intervention or Metformin. N Engl J Med. 2002;346(6):393-403.

4 5. Kinsinger LS, Jones KR, Kahwati L, et al. Design and dissemination of the MOVE! Weight-Management Program for Veterans. Prev Chronic Dis. 2009;6(3):A98.

6. Moyer VA. Screening for and management of obesity in adults: U.S. Preventive Services Task Force recommendation statement. Ann Intern Med. 2012;157(5):373-378

7. Jay M, Mateo KF, Horne M, Squires A, Kalet A, Sherman S. "In the military, your body and your life aren't your own:" Unique factors influencing health behavior change in overweight and obese veterans [abstract]. J Gen Intern Med. 2014;April(Supplemental 1).

8. Dahn JR, Fitzpatrick SL, Llabre MM, et al. Weight management for veterans: examining change in weight before and after MOVE! Obesity (Silver Spring). 2011;19(5):977-981.

9. VHA National Center for Health Promotion and Disease Prevention. MOVE! Weight Management Program for Veterans Fiscal Year 2013 Evaluation Report: Narrative Summary.; 2013. c

10. Whitlock E. Evaluating primary care behavioral counseling interventions An evidence-based approach. Am J Prev Med. 2002;22(4):267-284.

11. Damschroder LJ, Lutes LD, Kirsh S, et al. Small-changes obesity treatment among veterans: 12-month outcomes. Am J Prev Med. 2014;47(5):541-553.

12. Jay M, Gillespie C, Ark T, et al. Do Internists, Pediatricians, and Psychiatrists Feel Competent in Obesity Care? Using a Needs Assessment to Drive Curriculum Design. J Gen Intern Med. 2008;23(7):1066-1070.

13. Abid A, Galuska D, Khan LK, Gillespie C, Ford ES, Serdula MK. Are healthcare professionals advising obese patients to lose weight?: A trend analysis. Medscape Gen Med. 2005;7(4):10.

14. U.S. Department of Veteran Affairs, National Center for Health Promotion and Disease Prevention. MOVE! Implementation Best Practices: Evaluation Results, Final Report 2010.

15. . Maciejewski ML, Perkins M, Li Y-F, Chapko M, Fortney JC, Liu C-F. Utilization and expenditures of veterans obtaining primary care in community clinics and VA medical centers: an observational cohort study. BMC Health Serv Res. 2007;7(1):56.

16. Huang J, Yu H, Marin E, Brock S, Carden D, Davis T. Physicians' weight loss counseling in two public hospital primary care clinics. AAMC Acad Med J Assoc Am Med Coll. 2004;79(2):156-161.

17. Jay M, Chintapalli S, Oi KK, Squires A, Sherman S, Kalet A. Identifying barriers and facilitators to improving the implementation of weight management services within a patient-centered medical home [abstract]. J Gen Intern Med. 2014;April(Supplemental

12 18. Glasgow RE, Vogt TM, Boles SM. Evaluating the public health impact of health promotion interventions: the RE-AIM framework. Am J Public Health. 1999;89(9):1322-1327.

19. Yarnall KSH, Pollak KI, Ostbye T, Krause KM, Michener JL. Primary care: is there enough time for prevention? Am J Public Health. 2003;93(4):635-641.

20. . Ajzen I. Perceived Behavioral Control, Self-Efficacy, Locus of Control, and the Theory of Planned Behavior. J Appl Soc Psychol. 2002;32(4):665-683.

21. U.S. Department of Veteran Affairs and Department of Defense. Management of Obesity and Overweight (OBE) (2014). VA/DoD Clinical Practice Guideline for Screening and Management of Overweight and Obesity.

22. Kahwati LC, Lance TX, Jones KR, Kinsinger LS. RE-AIM evaluation of the Veterans Health Administration's MOVE! Weight Management Program. Transl Behav Med. 2011;1(4):551-560

23. Arigo D, Hooker S, Funderburk J, et al. Provider and staff perceptions of veterans' attrition from a national primary care weight management program. Prim Health Care Res Dev. 2014;(February):1-10. Rosland A-M, Nelson K, Sun H, et al. The patient-centered medical home in the Veterans Health Administration. Am J Manag Care. 2013;19(7):e263-e272.

24. Jacques L, Jensen TS, Schafer J, McClain S CJ. Decision Memo for Intensive Behavioral Therapy for Obesity (CAG-00423N).; 2011.

25. Ockene IS, Hebert JR, Ockene JK, et al. Effect of Physician-Delivered Nutrition Counseling Training and an Office-Support Program on Saturated Fat Intake, Weight, and Serum Lipid Measurements in a Hyperlipidemic Population: Worcester Area Trial for Counseling in Hyperlipidemia (WATCH). Vol 159. 1999.

26. Unrod M, Smith M, Spring B, DePue J, Redd W, Winkel G. Randomized Controlled Trial of a Computer-Based,

Tailored Intervention to Increase Smoking Cessation Counseling by Primary Care Physicians. J Gen Intern Med. 2007;22(4):478-484.

27. Goldstein MG, Whitlock EP, DePue J. Multiple behavioral risk factor interventions in primary care. Summary of research evidence. Am J Prev Med. 2004;27(2 Suppl):61-79.

28. Jay M, Gillespie CC, Schlair SL, et al. The impact of primary care resident physician training on patient weight loss at 12 months. Obesity (Silver Spring). 2013;21(1):45-50.

29. Nelson T, Jay M, Yin S, Squires A, Hung C, Altshuler L. Adventures in piloting an interprofessional obesity curriculum. J Gen Intern Med. 2014;April(Supplemental 1).

30. Bodenheimer T, Handley MA. Goal-setting for behavior change in primary care: an exploration and status report. Patient Educ Couns. 2009;76(2):174-180.

31. Damschroder LJ, Lutes LD, Kirsh S, et al. Small-changes obesity treatment among veterans: 12-month outcomes. Am J Prev Med. 2014;47(5):541-553.

32. Foster GD, Wadden TA, Makris AP, et al. Primary care physicians' attitudes about obesity and its treatment. Obes Res. 2003;11(10):1168-1177

33. Locke EA, Latham GP. Building a practically useful theory of goal setting and task motivation: A 35-year odyssey. Am Psychol. 2002;57(9):705-717.

34. Shilts MK, Horowitz M, Townsend MS. Goal setting as a strategy for dietary and physical activity behavior change: a review of the literature. Am J Heal Promot. 2004;19(2):81-93.

35. Levine DM, Savarimuthu S, Squires A, Nicholson J, Jay M. Technology-assisted weight loss interventions in primary care: a systematic review. J Gen Intern Med. 2015;30(1):107-117.

36. Mateo KF, Sikerwar S, Squires A, Kalet A, Sherman SE, Jay M. Development of a tailored, 5A's-based weight management intervention for Veterans within primary care. J Gen Intern Med. 2015;April(Supplemental 2).

37. Del Re AC, Maciejewski ML, Harris AHS. MOVE: weight management program across the Veterans Health Administration: patient- and facility-level predictors of utilization. BMC Health Serv Res. 2013;13:511.

38. Wolever RQ, Simmons LA, Sforzo GA, et al. A Systematic Review of the Literature on Health and Wellness Coaching: Defining a Key Behavioral intervention in Healthcare. Glob Adv Health Med. 2013;2(4):38-57.

39. Appel LJ, Clark JM, Yeh H-C, et al. Comparative Effectiveness of Weight-Loss Interventions in Clinical Practice. N Engl J Med. 2011;365(21):1959-1968.

40. Digenio AG, Mancuso JP, Gerber RA, Dvorak R V. Comparison of methods for delivering a lifestyle modification program for obese patients: a randomized trial. Ann Intern Med. 2009;150(4):255-262.

41. Garvin JT. Weight reduction goal achievement with high-intensity MOVE! treatment. Public Health Nurs. 2015;32(3):232-236.

42. Neuwirth EB, Schmittdiel JA, Tallman K, Bellows J. Understanding Panel Management: A comparative study of an emerging approach to population care. Perm J. 2007;11(3):12-20.

43. Schwartz MD, Jensen A, Wang B, et al. Panel Management to Improve Smoking and Hypertension Outcomes by VA Primary Care Teams: A Cluster-Randomized Controlled Trial. J Gen Intern Med. February 2015.

44. Peterson JC, Czajkowski S, Charlson ME, et al. Translating basic behavioral and social science research to clinical application: The EVOLVE mixed methods approach. Spec Issue Behav Med Clin Heal Psychol. 2013;81(2):217-230.

45. Czajkowski S, Powell L, Adler N, et al. From ideas to efficacy: The ORBIT model for developing behavioral treatments for chronic disease. Heal Psychol. 2015;Epub.

46. Perez HR, Nick MW, Mateo KF, Sherman SE, Kalet A, Jay M. "None of them apply to me": A usability study of the VA's MOVE!23 online weight management software in Latina Women [abstract]. J Gen Intern Med. 2014;April(Supplemental 1).

47. Gutnick D, Reims K, Davis C, Gainforth H, Jay M, Cole S. Brief action planning to facilitate behavior change and support self-management. J Clin Outcomes Manag. 2014;1(1).

48. U.S. Department of Veteran Affairs. MOVE! Coach [online]. 2015

49. U.S. Department of Veterans. MOVE! Coach app [online]. 2015.

50. . MyFitnessPal [online]. 2015.

51. Robertson C, Avenell A, Boachie C, et al. Should weight loss and maintenance programmes be designed differently for men? A systematic review of long-term randomised controlled trials presenting data for men and women: The ROMEO project. Obes Res Clin Pract. April 2015.

52. The Centre for Collaboration, Motivation and Innovation (CCMI) [online]. 2015

53. Wallace LS, Rogers ES, Roskos SE, Holiday DB, Weiss BD. Brief report: screening items to identify patients with limited health literacy skills. J Gen Intern Med. 2006;21(8):874-877.

54. U.S. Department of Veteran Affairs. MOVE! Coach [online]. 2015. Centers for Disease Control and Prevention. National Health and Nutrition Examination Survey (NHANES): Physical Activity Monitor (PAM) Procedures Manual [online].; 2011.

55. Friedberg JP, Rodriguez MA, Watsula ME, et al. Effectiveness of a tailored behavioral intervention to improve hypertension control: primary outcomes of a randomized controlled trial. Hypertension. 2015;65(2):440-446.

56. Kroenke K, Spitzer RL, Williams JB. The PHQ-9: validity of a brief depression severity measure. J Gen Intern Med. 2001;16(9):606-613.

57. Thompson FE, Midthune D, Sabar AF, Kahle LL, Schatzkin A, Kipnis V. Performance of a short tool to assess dietary intakes of fruits and vegetables, percentage energy from fat and fibre. Public Health Nutr. 2004;7(8):1097-1106.

58. Peterson ND, Middleton KR, Nackers LM, Medina KE, Milsom VA, Perri MG. Dietary self-monitoring and long-term success with weight management. Obesity (Silver Spring). 2014;22(9):1962-1967.

59. VHA National Center for Health Promotion and Disease Prevention. Fiscal Year 2011 Highlights.; 2011.

61. Centers for Disease Control and Prevention. Behavioral Risk Factor Surveillance System 2013 Questionnaire. Atlanta, Georgia; 2012.

62. Hagströmer M, Oja P, Sjöström M. the International Physical Activity Questionnaire (IPAQ): A study of concurrent and construct validity. Pub Health Nutr. 2006;9(6):755-62.

63. Murphy SL. Review of physical activity measurement using accelerometers in older adults: considerations for research design and conduct. Prev Med (Baltim). 2009;48(2):108-114.
