# Supplementary material for: Peer Coaching to Support Weight Management in Primary Care: A Cluster Randomized Clinical Trial
Source: JAMA Netw Open. 2025 Sep 2;8(9):e2529136. doi: 10.1001/jamanetworkopen.2025.29136 (PMC12406063; doi:10.1001/jamanetworkopen.2025.29136)
Supplement: Supplement 2. — eMethods 1. Participant Inclusion/Exclusion Criteria eMethods 2. Recruitment Procedure eMethods 3. PAL Intervention Description eMethods 4. Clinical and Behavioral Outcome Measures and Procedures eMethods 5. Sample Size Calculation eMethods 6. Exploratory Analyses eFigure. Primary Outcome: Mean Weight Change in kg by Patient Characteristic and Engagement With Intervention eTable 1. Baseline Socioeconomic Characteristics and Engagement With Intervention by Race and Ethnicity Group eTable 2. Baseline Socioeconomic Characteristics and Engagement With Intervention by Gender Group eTable 3. Baseline Socioeconomic Characteristics and Engagement With Intervention by Age Group eTable 4. Sensitivity Analyses of Clinical Outcomes at 6 and 12 Months by Treatment Arm (Excluding Patients Using Obesity Medications) eTable 5. Sensitivity Analyses of Behavioral Outcomes at 6 and 12 Months by Treatment Arm (Excluding Patients Using Obesity Medications) eReferences [file jamanetwopen-e2529136-s002.pdf]

Supplemental Online Content

Wittleder S, Wong L, Ruan AM, et al. Peer coaching to support weight management in primary care: a cluster randomized clinical trial. *JAMA Netw Open*. 2025;8(9):e2529136. doi:10.1001/jamanetworkopen.2025.29136

eMethods 1. Participant Inclusion/Exclusion Criteria
eMethods 2. Recruitment Procedure
eMethods 3. PAL Intervention Description
eMethods 4. Clinical and Behavioral Outcome Measures and Procedures
eMethods 5. Sample Size Calculation
eMethods 6. Exploratory Analyses
eFigure. Primary Outcome: Mean Weight Change in kg by Patient Characteristic and Engagement With Intervention
eTable 1. Baseline Socioeconomic Characteristics and Engagement With Intervention by Race and Ethnicity Group
eTable 2. Baseline Socioeconomic Characteristics and Engagement With Intervention by Gender Group
eTable 3. Baseline Socioeconomic Characteristics and Engagement With Intervention by Age Group
eTable 4. Sensitivity Analyses of Clinical Outcomes at 6 and 12 Months by Treatment Arm (Excluding Patients Using Obesity Medications)
eTable 5. Sensitivity Analyses of Behavioral Outcomes at 6 and 12 Months by Treatment Arm (Excluding Patients Using Obesity Medications)
eReferences

This supplemental material has been provided by the authors to give readers additional information about their work.

© 2025 Wittleder S et al. *JAMA Network Open*

eMethods 1: Participant inclusion/exclusion criteria

Inclusion criteria: (1) 18 to 69 years of age; (2) BMI $\geq$ 30 kg/m^2 (obesity) or $\geq$ 25 kg/m^2 (overweight) with a weight-associated comorbidity (i.e., hypertension, hyperlipidemia, osteoarthritis, metabolic syndrome (defined by AHA/NHLBI criteria), or pre-diabetes)[1]; (3) had been under the care of a primary care physician (PCP) with at least one previous VHA visit within the past two years. Additionally, eligible patients had access to a telephone and were able to travel to the Brooklyn VA. Exclusion criteria included participants who were not part of the Veteran population, had a documented history of active psychosis, and those with other cognitive issues. Additional exclusionary factors included having more than four MOVE! Program sessions in the previous year, pregnancy, having their PCP state they should not participate, or having a self-reported inability to read at least at a fifth-grade level.

© 2025 Wittleder S et al. *JAMA Network Open*

eMethods 2: Recruitment

Potentially eligible patients of participating PCPs were identified via extracted lists through the Veteran Health Information Systems and Technology Architecture. Lists of patients were sent to PCPs who had the option to notify research assistants (RAs) not to contact those they deemed inappropriate for participation. RAs then sent potentially eligible patients a letter with information about the study and then contacted patients by phone to screen for interest and eligibility. Participants in both arms could receive up to $155 for completing study assessments. Enrollment began in February 2018 and was terminated prematurely in March 2020 due to the COVID-19 pandemic

eMethods 3: PAL Intervention Description

During their baseline visit, patients used an online tool to assess their health behaviors, identify barriers, and set personalized goals for weight loss, diet, and physical activity, aligning with the first 'A' (Assess). This tool generated a summary report, which included personalized advice and tailored educational handouts, that guided peer coaches in providing specific feedback, helping set realistic goals, and facilitating behavior change, addressing the next three 'As' (Advise, Agree, and Assist). Peer coaches employed motivational interviewing (MI) and SMART (Specific, Measurable, Attainable, Relevant, Timely) goal-setting techniques. Patients received education on self-monitoring behaviors and tools, such as pedometers and food logs, facilitating ongoing engagement with their health goals. Peer coaches provided information about the MOVE! program and encouraged patients to enroll.

Over the course of the intervention, patients received up to 12 telephone coaching calls, administered by peer coaches at biweekly intervals during the first month and then monthly for the duration of the intervention. These calls served to reinforce goal adherence, provide counseling, encourage incremental behavior changes, address barriers to care and social determinants of health (e.g., connecting Veterans to services to address homelessness or food insecurity), and support enrollment and engagement in MOVE! or other intensive weight management programs.

Importantly, the online tool also generated a template to guide and document health coach counseling and it was entered into the VA's Electronic Health Record (EHR) system to communicate with the healthcare team, thus fulfilling the fifth 'A' (Arrange) by arranging for continuity of care and support. PCPs in the PAL intervention arm received brief 5As training and clinical reminders to facilitate weight management counseling. PCPs in PAL were instructed to provide brief 5As counseling and MI during regular PC visits and were encouraged to work collaboratively with the peer coaches who used a toolkit to refer to VA services and communicate with the PCPs as needed to address barriers to weight management (e.g. mental health challenges, food insecurity). Through these coordinated efforts, PAL offers a comprehensive and personalized approach to obesity counseling, aiming to empower patients towards sustained lifestyle changes.

<u>Peer Coach and PCPs Training and Fidelity</u>

Peer coaches were Veterans who had a bachelor’s degree and had received honorable discharge; none had a clinical degree or license. They received at least 20 hours of training on the 5As counseling framework, MIand brief action planning. Training activities involved reviewing manuals, role-playing, and analyzing audiotapes, with materials adapted from the Centre for Collaboration, Motivation, and Innovation.[2,3] They were trained to use a toolkit that contained VA- and city-wide resources for Veterans. Peer coaches convened biweekly for continuous development, discussing cases and exchanging best practices. Fidelity to intervention protocols was ensured through the use of an adapted VA ASPIRE health coach fidelity checklist[4], with at least 10% of coaching sessions recorded and assessed to maintain skill acquisition and counseling quality.

PCPs received a brief (45 mins), targeted training in the first year of the study, with at least one follow-up session by the Principal Investigator (PI). This training covered the 5As, Brief MI protocols, PAL intervention components, and working with peer coaches.

© 2025 Wittleder S et al. *JAMA Network Open*

eMethods 4: Clinical and Behavioral Outcome Measures and Procedures

Height and Weight. RAs measured height once with a SECA 213 Portable stadiometer to the nearest 0.5 cm. Weight was measured twice using a HealthOMeter 349KLX Digital Medical Scale and rounded to the nearest 0.1 pounds. A third weight measurement was taken only if the first two measurements differed by 0.5 lbs or more. For patients that had a third measurement taken, the two closest values were averaged.

Waist Circumference. RAs measured waist circumference on exposed skin with minimal respiration. The measurements were conducted at the highest point of the iliac crest and rounded down to the nearest 0.25 inch. Two measurements of waist circumference were obtained, but if the initial two readings exhibited a difference of 0.5 inches or greater, a third measurement was taken. The average of the two closest values was then used for data analysis.

Blood Pressure. RAs utilized an automated sphygmomanometer (Omron HEM-907XL) to measure blood pressure. This assessment occurred at least 30 minutes into the survey administration during visits, considering any potential influence of caffeine intake from physical activity. Blood pressure readings were taken twice, and if the initial two systolic or diastolic values exhibited a difference of 5.0 mm Hg or more, RAs repeated the measurements. The average of the two closest values was then used for data analysis.

Weight Management Program Attendance. Patients reported whether they had attended a weight management program in the past 6 months such as MOVE! or TeleMove!, Telephone Lifestyle Coaching (TLC), Diabetes prevention program (DPP), Weight Watchers, Overeater Anonymous at least once in the past 6 months. We created a binary indicator for having attended any weight management program (1) or not (0).

Physical Activity Assessment. RAs administered the International Physical Activity Questionnaire-Short Form (IPAQ-SF)[5,6], which measures the frequency (in days) and duration (in minutes) of walking, moderate-, and vigorous-intensity physical activity during the last 7 days. The IPAQ-SF yields small-to-moderate correlation with device-based activity monitoring.[5] We truncated walking, moderate, and vigorous durations to maximal 180 minutes and set days with < 10 min of activity to 0, consistent with the IPAQ scoring protocol.[7] We summed walking, moderate, and vigorous minutes per week into continuous scores each.

Metabolic Equivalents (METs)-minutes were computed, representing the multiplicative value of energy expended carrying out physical activity beyond the 1 MET expended at rest (i.e., 3.3 for walking, 4 for moderate activity, and 8 for vigorous activity). We summed walking, moderate, and vigorous MET-minutes/week into a continuous total MET-minutes/week score.

Dietary Assessments. Fruit and Vegetable Intake: Fruit and vegetable intake was assessed using the six-item subscale of the Food Behavior Checklist (FBC).[8] The FBC is considered a valid and reliable measure due to its rigorous development process and strong psychometric properties, which have been demonstrated in the evaluation of community nutrition interventions aimed at improving dietary habits and food security among diverse populations.[9] Patients reported how much fruit and vegetables they consumed per day, with the scale options: *None* (1), *0.5 cup* (1.5), *1 cup* (2), *1.5 cups* (2.5), *2 cups* (3), *2.5 cups* (3.5), and *3 cups or more* (4). Patients further indicated if they ate fruits and vegetables as snacks, had more than one type of fruits or vegetables per day, and, if they had two or more kinds of vegetable with their main meal. Scale options (coded value) for these questions were: *No* (1), *Yes, sometimes (*2), *Yes, often* (3), and *Yes, always* (4). A mean score was calculated (α = .79), with higher numbers indicating more/healthier fruits and vegetables intake (possible range 1-4).[8]

Healthy Dietary Behaviors: Healthy dietary behaviors were assessed using the six-item subscale from the Latino Dietary Behaviors Questionnaire (LDBQ). [10] Patients reported how often they consumed soda, fried foods, regular white rice or bread with the scale options: *Never* (5), *Less than once a week* (4), *About once a week* (3), *2-5 times per week* (2), *About once a day* (1), *2 or more times per day* (0). Patients further indicated how often they ate at fast food restaurants, with the scale options: *Almost never or less than one time per months* (3), *One time per month* (2), *2-3 times per month* (1), *3 or more times per month* (0). Finally, patients indicated how often they controlled portions, or opted for healthier alternatives. Available answer choices were *Rarely or never* (0), *Sometimes* (1), *Many times* (2), *All of the time* (3). A sum score was calculated (α = .57), with higher numbers indicating healthier eating behaviors (possible range 0-24).

Sweet and Salty Snack Intake: Sweet and salty snack intake was assessed using two items from the Rapid Eating Assessment–Shortened Version (REAP-S).[11] The REAP-S is considered a valid and reliable tool, shown by its strong correlations with diet quality indices that provides a comprehensive evaluation of dietary habits.[12] Patients reported how often they ate salty snacks or sweets more than 2 times per day, with the scale options: *usually/often* (1), *sometimes* (2), and *rarely/never* (3). A sum score was calculated, with higher scores indicating healthier (less frequent) snack intake (possible range 2-6).

COVID-19 protocol changes. To accommodate COVID-19 pandemic restrictions, protocols for follow-up data were modified. Staff mailed scales and tape measures to patients' homes and recorded clinical measures and

© 2025 Wittleder S et al. *JAMA Network Open*

surveys via phone. Due to logistical constraints, the study team was unable to provide remote blood pressure machines and accelerometers, thus these measures were discontinued. Adverse events were assessed via participant self-report and electronic health records.

eMethods 5: Sample Size Calculation

We performed sample size calculations to test our primary hypothesis at $\alpha = 0.05$ significance level (2-sided) and 80% power, for the primary outcome of weight change (in kilogram) between the two arms at 12 months follow-up. We assumed a small, but conservative, intra-class correlation coefficient of 0.03[13] for patients of each cluster and a coefficient of variation of 0.25 for the cluster size, which was based on the data available at the Brooklyn VA. With 369 evaluable patients, we would be able to detect a mean 2.4 (6.0) kg difference between intervention and comparison arms, which is in line with findings from the ASPIRE VA study[14] and a systematic review.[15] We initially planned to recruit 461 patients, assuming a 20% drop out rate. Given that the study ended early, we did not achieve this enrollment goal.

© 2025 Wittleder S et al. *JAMA Network Open*

eMethods 6: Exploratory Analyses

Exploratory analyses were conducted to identify patient characteristics (gender, race, age) and use of intervention components (weight management program attendance and completed coaching calls), which may have influenced intervention effectiveness. We estimated the mean weight change (in kilogram) at six and 12 months within each level, stratified by treatment arm to examine differences in weight trends. For gender, race, and age, we also examined the distribution of baseline socio-demographics and the use of intervention components within each level. These analyses were descriptive in nature and used data only from patients with available data. We conducted additional sensitivity analyses that removed two patients who received anti-obesity medications during the study period and found that they were similar to the primary study conclusions

eFigure. Primary outcome: Mean weight change in kg by patient characteristic and engagement with intervention.

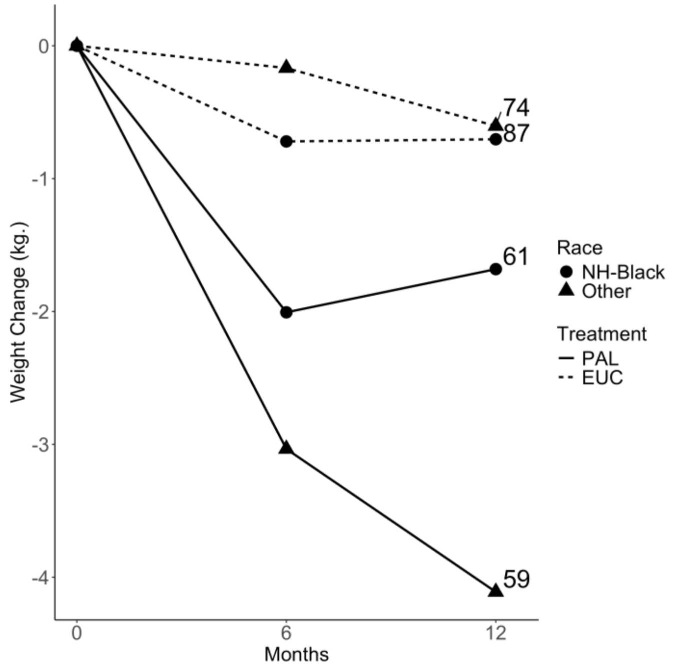

Panel A: Weight Change according to race/ethnicity

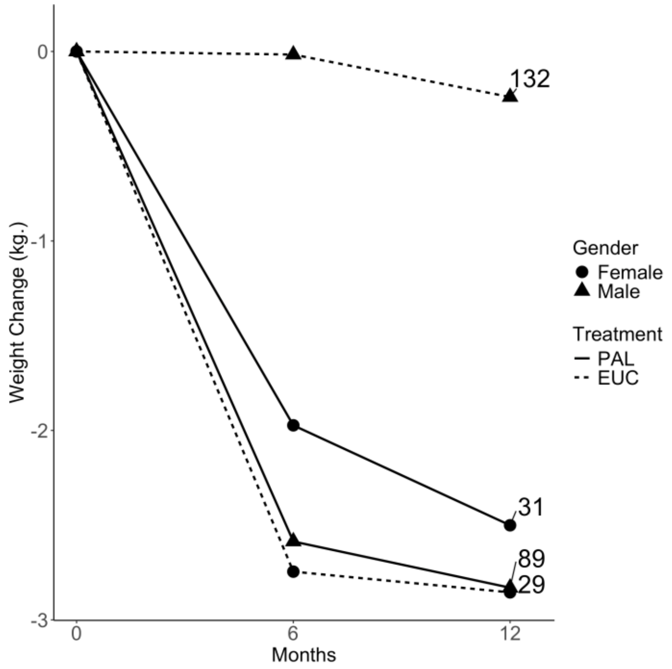

Panel B: Weight Change according to gender

© 2025 Wittleder S et al. *JAMA Network Open*

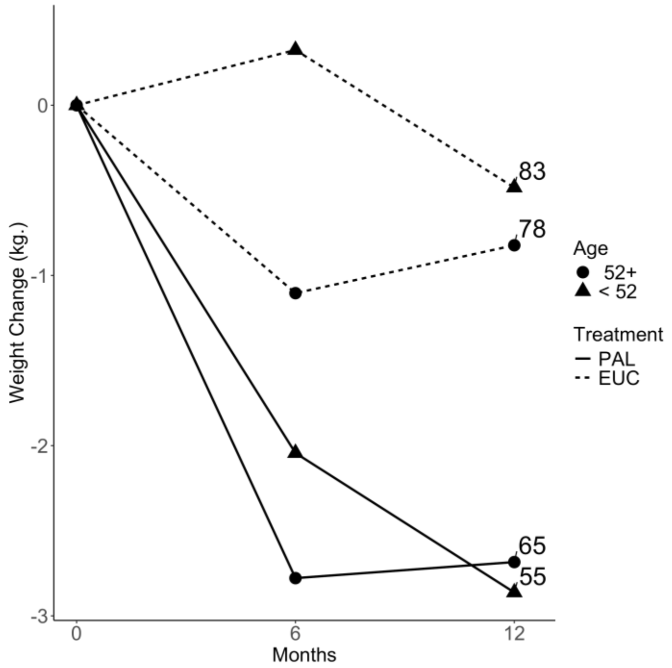

Panel C: Weight Change according age

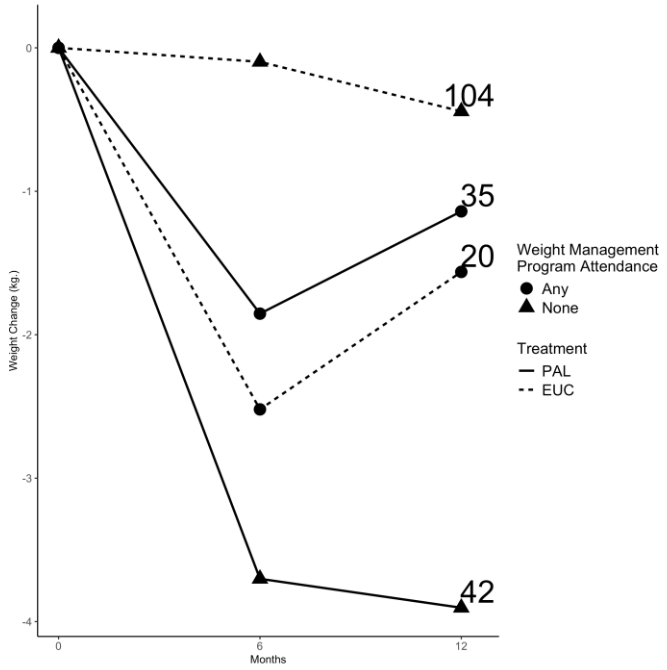

Panel D: Weight Change according to weight management program attendance

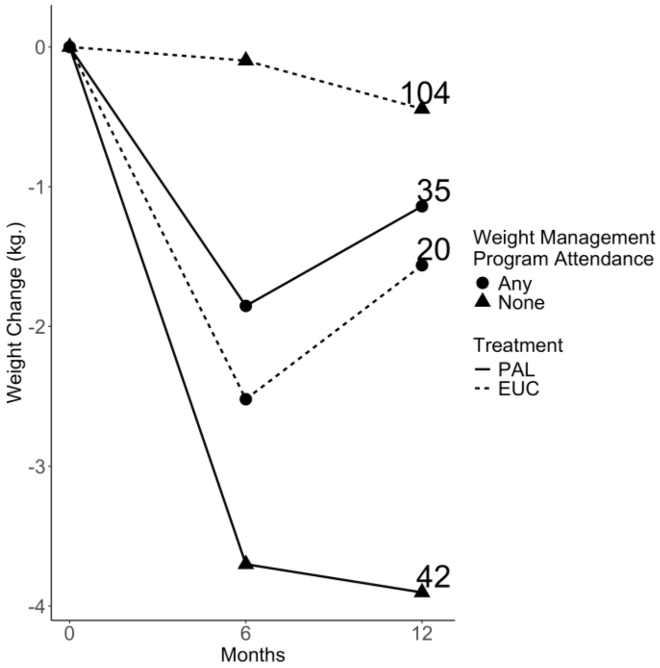

Panel E: Weight Change according to number of completed calls in the PAL intervention arm

Note. Shown is the weight change in kg over time according to race/ethnicity: NH-Black vs. other (Panel A), gender: Male vs. female (Panel B), age: Below 52 years vs. ≥ 52 years (Panel C), weight management program attendance: any vs. none (Panel D), and number of completed calls in the PAL intervention arm (Panel E). NH-Black was compared to all other races/ethnicities categories combined: Hispanic, Non-Hispanic Other, and Non-Hispanic White. Age cut at 52 years was the median age in our sample. Weight management attendance groups indicate if the patient reported having attended any weight management program (any) or not (none). Among patients who completed at least one call, the median number of completed calls was five. Groupings correspond to patients completing zero calls, and those with at least one call falling below this median and at least the median. Numbers at the end of each line represent *n* within each subgroup.

© 2025 Wittleder S et al. *JAMA Network Open*

eTable 1. Baseline socioeconomic characteristics and engagement with intervention by race and ethnicity group

Characteristic	Overall (N=281)	EUC: All other race/ethnic groups (n=74)	PAL: All other race/ethnic groups (n=59)	EUC: NH-Black (n=87)	PAL: NH-Black (n=61)	*p* value	*Missing (%)*
**Age, mean (SD), y**	50.6 (11.5)	49.0 (13.0)	50.7 (12.7)	50.7 (10.3)	52.6 (9.7)	0.35	0.0
**Gender, No. (%)**						0.24	0.0
Female	60 (21.4)	10 (13.5)	15 (25.4)	19 (21.8)	16 (26.2)		
Male	221 (78.6)	64 (86.5)	44 (74.6)	68 (78.2)	45 (73.8)		
**Race/Ethnicity, No. (%)[a]**						**<0.01**	0.0
Hispanic	61 (21.7)	38 (51.4)	23 (39.0)	0 (0.0)	0 (0.0)		
Non-Hispanic Black	148 (52.7)	0 (0.0)	0 (0.0)	87 (100.0)	61 (100.0)		
Non-Hispanic Other	24 (8.5)	10 (13.5)	14 (23.7)	0 (0.0)	0 (0.0)		
Non-Hispanic White	48 (17.1)	26 (35.1)	22 (37.3)	0 (0.0)	0 (0.0)		
**Born in United States, No. (%)**						0.12	0.0
Yes	194 (69.0)	55 (74.3)	46 (78.0)	55 (63.2)	38 (62.3)		
No	87 (31.0)	19 (25.7)	13 (22.0)	32 (36.8)	23 (37.7)		
**English at home, No. (%)**						**<0.01**	0.7
Yes	262 (93.9)	63 (86.3)	52 (89.7)	87 (100.0)	60 (98.4)		
No	17 (6.1)	10 (13.7)	6 (10.3)	0 (0.0)	1 (1.6)		
**Employment Status, No. (%)[b]**						0.38	0.4
Employed	159 (56.8)	46 (62.2)	30 (50.8)	52 (60.5)	31 (50.8)		
Unemployed	121 (43.2)	28 (37.8)	29 (49.2)	34 (39.5)	30 (49.2)		
**Marital Status, No. (%)[c]**						0.48	0.0
Married or in a marriage-like relationship	158 (56.2)	37 (50.0)	32 (54.2)	54 (62.1)	35 (57.4)		
Single/Never Married/Separated/ Divorced/Widowed	123 (43.8)	37 (50.0)	27 (45.8)	33 (37.9)	26 (42.6)		
**Educational Attainment, No. (%)[d]**						0.64	0.0
High School Graduate or Less	58 (20.6)	16 (21.6)	14 (23.7)	14 (16.1)	14 (23.0)		
More than High School	223 (79.4)	58 (78.4)	45 (76.3)	73 (83.9)	47 (77.0)		
**Depression Risk (CES-D-SF ≥ 8, No. (%)[e]**						0.48	0.4
Yes	68 (24.3)	19 (25.7)	16 (27.6)	16 (18.4)	17 (27.9)		
No	212 (75.7)	55 (74.3)	42 (72.4)	71 (81.6)	44 (72.1)		

© 2025 Wittleder S et al. *JAMA Network Open*

Characteristic	Overall (N=281)	EUC: All other race/ethnic groups (n=74)	PAL: All other race/ethnic groups (n=59)	EUC: NH-Black (n=87)	PAL: NH-Black (n=61)	*p* value	*Missing (%)*
**Clinical and Behavioral Measures**							
**Height, mean (SD), centimeter**	172.8 (8.8)	171.9 (8.4)	169.5 (8.4)	175.4 (8.4)	173.4 (9.1)	**<0.01**	0.0
**Weight, mean (SD), kilogram**	99.9 (18.2)	97.5 (16.8)	94.9 (15.7)	103.2 (15.6)	102.8 (23.6)	**0.02**	0.0
**BMI, mean (SD), kg/m^2**	33.4 (5.1)	32.9 (4.7)	32.9 (3.9)	33.7 (4.6)	34.1 (6.9)	0.44	0.0
**Waist circumference, mean (SD), in**	42.8 (5.4)	42.7 (4.9)	41.8 (5.6)	42.8 (4.5)	43.9 (6.7)	0.20	0.0
**Diastolic blood pressure, mean (SD), mmHg**	78.5 (19.9)	78.0 (11.2)	77.1 (10.6)	77.7 (10.0)	81.4 (37.8)	0.63	0.7
**Systolic blood pressure, mean (SD), mmHg**	125.6 (33.1)	130.5 (59.6)	123.6 (14.0)	123.8 (14.9)	123.9 (14.9)	0.52	0.7
**Walking Physical Activity, mean (SD), minutes/week[f]**	350.6 (383.3)	401.8 (449.6)	340.8 (365.8)	332.7 (369.1)	323.4 (332.6)	0.60	0.0
**Moderate Physical Activity, mean (SD), minutes/week[f]**	177.1 (278.3)	185.7 (271.7)	151.0 (248.7)	185.0 (291.0)	180.9 (299.7)	0.88	0.7
**Vigorous Physical Activity, mean (SD), minutes/week[f]**	204.0 (284.8)	204.7 (277.8)	207.4 (288.7)	220.3 (287.7)	176.6 (290.2)	0.84	0.0
**Total Physical Activity, mean (SD), MET minutes/week[f]**	3511.0 (3570.1)	3745.3 (3765.4)	3387.7 (3385.3)	3600.5 (3663.0)	3217.2 (3427.5)	0.84	0.7
**Fruit and Vegetable Intake, mean (SD)[g]**	2.4 (1.3)	2.2 (1.3)	2.7 (1.2)	2.3 (1.3)	2.5 (1.2)	0.15	0.0
**Healthy Dietary Behaviors, mean (SD)[h]**	13.2 (3.4)	13.2 (3.6)	12.8 (3.3)	13.0 (3.4)	14.0 (3.3)	0.21	0.4
**Sweet and Salty Snack Intake, mean (SD)[i]**	4.5 (1.2)	4.6 (1.2)	4.4 (1.2)	4.7 (1.2)	4.4 (1.1)	0.39	0.0
**Use of weight management programs and PAL**							
**Weight Management Program Attendance, %**						**<0.01**	27.0
Any	55 (26.8)	7 (12.3)	13 (34.2)	15 (21.4)	20 (50.0)		
None	150 (73.2)	50 (87.7)	25 (65.8)	55 (78.6)	20 (50.0)		
**Coaching calls completed, mean (SD)**	4.4 (3.0)	-	4.2 (3.2)	-	4.6 (2.8)	0.41	0.0

Abbreviations: BMI, body mass index; CES-D-SF, Center for Epidemiologic Studies Depression Scale Short-Form; EUC, enhanced usual care; mmHg, millimeters of mercury; MET, metabolic equivalent; PAL, Peer Assisted Lifestyle; SMD, standardized mean difference

© 2025 Wittleder S et al. *JAMA Network Open*

[a] Race and ethnicity were self-reported by participants; Other races and ethnicities (n=7); Asian (n=6), American Indian or Alaskan Native (n=8), Native-Hawaiian/Pacific Islander (n=3) were included in the other category because of small cell sizes.
[b] Employed included working full time (n=144) and working part time (n=15). Unemployed included unemployed or laid off (n=14), looking for work (n=5), student (n=19), keeping house or raising children full-time (n=4), and retired (n=66).
[c] Marital Status included Single/Never Married (n=79), Married or in a marriage-like relationship (n=123), Separate (n=15), Divorced (n=59), and Widowed (n=5).
[d] Education Status included Never attended school or only kindergarten (n = 0), Grades 1 through 8 (Elementary) (n = 1), Grades 9 through 11 (n = 0), Grade 12 or GED (n = 57), Associate's Degree (n = 48), Some college (n = 64), College 4 years or more (n = 71), Some graduate or professional training (n = 8), and Graduate or professional degree (n = 30).
[e] CES-D-SF is a seven-item screener for suspected major depressive disorder in US community studies (cutoff score ≥8).
[f] We truncated walking, moderate, and vigorous durations to maximal 180 minutes and set days with < 10 minutes of activity to 0, consistent with the Physical Activity Questionnaire-Short Form scoring protocol. MET minutes were computed, representing the multiplicative value of energy expended carrying out physical activity >1 MET expended at rest (i.e., 3.3 for walking, 4 for moderate activity, and 8 for vigorous activity). We summed walking, moderate, and vigorous MET-minutes/week into a continuous total MET-minutes/week score.
[g] Fruit and Vegetable Intake was assessed using the six-item subscale of the Food Behavior Checklist. Scores could range from 1-4, with higher numbers indicating more/healthier fruits and vegetables intake.
[h] Healthy Dietary Behaviors were assessed using the six-item subscale from the Latino Dietary Behaviors Questionnaire. Scores could range from 0-24, with higher numbers indicating healthier eating behaviors.
[i] Sweet and Salty Snack Intake was assessed using two items from the Rapid Eating Assessment–Shortened Version. Scores could range from 2-6, with higher scores indicating healthier (less frequent) snack intake.

© 2025 Wittleder S et al. *JAMA Network Open*

eTable 2. Baseline socioeconomic characteristics and engagement with intervention by gender group

Characteristic	Overall (N=281)	EUC: Female (n=29)	PAL: Female (n=31)	EUC: Male (n=132)	PAL: Male (n=89)	*p* value	*Missing (%)*
**Age, mean (SD), y**	50.6 (11.5)	50.8 (11.6)	46.1 (11.9)	49.7 (11.7)	53.6 (10.5)	0.01	0.0
**Gender, No. (%)**							
Female	60 (21.4)	29 (100.0)	31 (100.0)	0 (0.0)	0 (0.0)	<0.01	0.0
Male	221 (78.6)	0 (0.0)	0 (0.0)	132 (100.0)	89 (100.0)		
**Race/Ethnicity, No. (%)**[a]							
Hispanic	61 (21.7)	3 (10.3)	7 (22.6)	35 (26.5)	16 (18.0)	0.52	0.0
Non-Hispanic Black	148 (52.7)	19 (65.5)	16 (51.6)	68 (51.5)	45 (50.6)		
Non-Hispanic Other	24 (8.5)	2 (6.9)	4 (12.9)	8 (6.1)	10 (11.2)		
Non-Hispanic White	48 (17.1)	5 (17.2)	4 (12.9)	21 (15.9)	18 (20.2)		
**Born in United States, No. (%)**							
Yes	194 (69.0)	19 (65.5)	24 (77.4)	91 (68.9)	60 (67.4)	0.73	0.0
No	87 (31.0)	10 (34.5)	7 (22.6)	41 (31.1)	29 (32.6)		
**English at home, No. (%)**							
Yes	262 (93.9)	28 (96.6)	28 (93.3)	122 (93.1)	84 (94.4)	0.91	0.7
No	17 (6.1)	1 (3.4)	2 (6.7)	9 (6.9)	5 (5.6)		
**Employment Status, No. (%)**[b]							
Employed	159 (56.8)	17 (58.6)	17 (54.8)	81 (61.8)	44 (49.4)	0.33	0.4
Unemployed	121 (43.2)	12 (41.4)	14 (45.2)	50 (38.2)	45 (50.6)		
**Marital Status, No. (%)**[c]							
Married or in a marriage-like relationship	158 (56.2)	23 (79.3)	23 (74.2)	68 (51.5)	44 (49.4)	<0.01	0.0
Single/Never Married/Separated/ Divorced/Widowed	123 (43.8)	6 (20.7)	8 (25.8)	64 (48.5)	45 (50.6)		
**Educational Attainment, No. (%)**[d]							
High School Graduate or Less	58 (20.6)	4 (13.8)	4 (12.9)	26 (19.7)	24 (27.0)	0.24	0.0
More than High School	223 (79.4)	25 (86.2)	27 (87.1)	106 (80.3)	65 (73.0)		
**Depression Risk (CES-D-SF ≥ 8, No. (%)**[e]							
Yes	68 (24.3)	4 (13.8)	14 (45.2)	31 (23.5)	19 (21.6)	0.02	0.4
No	212 (75.7)	25 (86.2)	17 (54.8)	101 (76.5)	69 (78.4)		
**Clinical and Behavioral Measures**							

© 2025 Wittleder S et al. *JAMA Network Open*

Characteristic	Overall (N=281)	EUC: Female (n=29)	PAL: Female (n=31)	EUC: Male (n=132)	PAL: Male (n=89)	*p* value	*Missing (%)*
**Height, mean (SD), centimeter**	172.8 (8.8)	165.4 (6.9)	163.5 (6.9)	175.7 (7.7)	174.3 (7.8)	<0.01	0.0
**Weight, mean (SD), kilogram**	99.9 (18.2)	91.7 (15.1)	86.4 (12.6)	102.5 (16.0)	103.3 (20.9)	<0.01	0.0
**BMI, mean (SD), kg/m^2**	33.4 (5.1)	34.0 (5.0)	32.2 (3.2)	33.2 (4.6)	33.9 (6.2)	0.34	0.0
**Waist circumference, mean (SD), in**	42.8 (5.4)	40.2 (3.8)	39.2 (4.7)	43.3 (4.7)	44.2 (6.2)	<0.01	0.0
**Diastolic blood pressure, mean (SD), mmHg**	78.5 (19.9)	79.8 (11.8)	76.2 (7.4)	77.4 (10.2)	80.4 (32.1)	0.63	0.7
**Systolic blood pressure, mean (SD), mmHg**	125.6 (33.1)	123.6 (15.3)	120.6 (16.1)	127.7 (45.8)	124.8 (13.7)	0.72	0.7
**Walking Physical Activity, mean (SD), minutes/week[f]**	350.6 (383.3)	420.0 (428.6)	368.2 (349.0)	352.3 (404.3)	319.4 (348.7)	0.66	0.0
**Moderate Physical Activity, mean (SD), minutes/week[f]**	177.1 (278.3)	103.3 (167.8)	146.5 (307.7)	203.5 (298.3)	172.6 (264.4)	0.31	0.7
**Vigorous Physical Activity, mean (SD), minutes/week[f]**	204.0 (284.8)	170.0 (261.7)	88.2 (136.1)	222.6 (286.8)	227.8 (318.3)	0.08	0.0
**Total Physical Activity, mean (SD), MET minutes/week[f]**	3511.0 (3570.1)	3159.1 (3654.0)	2516.4 (2369.7)	3778.9 (3713.4)	3566.5 (3648.0)	0.34	0.7
**Fruit and Vegetable Intake, mean (SD)[g]**	2.4 (1.3)	2.8 (1.6)	2.7 (1.3)	2.2 (1.2)	2.6 (1.2)	0.02	0.0
**Healthy Dietary Behaviors, mean (SD)[h]**	13.2 (3.4)	12.3 (3.8)	13.1 (3.5)	13.3 (3.4)	13.5 (3.3)	0.47	0.4
**Sweet and Salty Snack Intake, mean (SD)[i]**	4.5 (1.2)	4.7 (1.2)	4.6 (0.8)	4.6 (1.2)	4.3 (1.2)	0.24	0.0
**Use of weight management programs and PAL**							
**Weight Management Program Attendance, %**							
Any	55 (26.8)	7 (38.9)	9 (56.2)	15 (13.8)	24 (38.7)	<0.01	27.0
None	150 (73.2)	11 (61.1)	7 (43.8)	94 (86.2)	38 (61.3)		
**Coaching calls completed, mean (SD)**	4.4 (3.0)	-	3.9 (2.7)	-	4.6 (3.1)	0.32	0.0

Abbreviations: BMI, body mass index; CES-D-SF, Center for Epidemiologic Studies Depression Scale Short-Form; EUC, enhanced usual care; mmHg, millimeters of mercury; MET, metabolic equivalent; PAL = Peer Assisted Lifestyle; SMD, standardized mean difference

[a] Race and ethnicity were self-reported by participants; Other races and ethnicities (n=7), Asian (n=6), American Indian or Alaskan Native (n=8), Native-Hawaiian/Pacific Islander (n=3) were included in the other category because of small cell sizes.

[b] Employed included working full time (n=144) and working part time (n=15). Unemployed included unemployed or laid off (n=14), looking for work (n=5), student (n=19), keeping house or raising children full-time (n=4), and retired (n=66).

[c] Marital Status included Single/Never Married (n=79), Married or in a marriage-like relationship (n=123), Separate (n=15), Divorced (n=59), and Widowed (n=5).

© 2025 Wittleder S et al. *JAMA Network Open*

[d] Education Status included Never attended school or only kindergarten (n = 0), Grades 1 through 8 (Elementary) (n = 1), Grades 9 through 11 (n = 0), Grade 12 or GED (n = 57), Associate's Degree (n = 48), Some college (n = 64), College 4 years or more (n = 71), Some graduate or professional training (n = 8), and Graduate or professional degree (n = 30).
[e] CES-D-SF is a seven-item screener for suspected major depressive disorder in US community studies (cutoff score ≥8).
[f] We truncated walking, moderate, and vigorous durations to maximal 180 minutes and set days with < 10 minutes of activity to 0, consistent with the Physical Activity Questionnaire-Short Form scoring protocol. MET minutes were computed, representing the multiplicative value of energy expended carrying out physical activity >1 MET expended at rest (i.e., 3.3 for walking, 4 for moderate activity, and 8 for vigorous activity). We summed walking, moderate, and vigorous MET-minutes/week into a continuous total MET-minutes/week score.
[g] Fruit and Vegetable Intake was assessed using the six-item subscale of the Food Behavior Checklist. Scores could range from 1-4, with higher numbers indicating more/healthier fruits and vegetables intake.
[h] Healthy Dietary Behaviors were assessed using the six-item subscale from the Latino Dietary Behaviors Questionnaire. Scores could range from 0-24, with higher numbers indicating healthier eating behaviors.
[i] Sweet and Salty Snack Intake was assessed using two items from the Rapid Eating Assessment–Shortened Version. Scores could range from 2-6, with higher scores indicating healthier (less frequent) snack intake.

© 2025 Wittleder S et al. *JAMA Network Open*

eTable 3. Baseline socioeconomic characteristics and engagement with intervention by age group

Characteristic	Overall (N=281)	EUC: 52+ (n=78)	PAL: 52+ (n=65)	EUC: < 52 (n=83)	PAL: < 52 (n=55)	*p* value	*Missing (%)*
**Age, mean (SD), y**	50.6 (11.5)	59.8 (5.2)	60.4 (5.0)	40.6 (7.5)	41.3 (7.2)	<0.01	0.0
**Gender, No. (%)**							
Female	60 (21.4)	15 (19.2)	13 (20.0)	14 (16.9)	18 (32.7)	0.14	0.0
Male	221 (78.6)	63 (80.8)	52 (80.0)	69 (83.1)	37 (67.3)		
**Race/Ethnicity, No. (%)[a]**							
Hispanic	61 (21.7)	15 (19.2)	10 (15.4)	23 (27.7)	13 (23.6)	0.36	0.0
Non-Hispanic Black	148 (52.7)	45 (57.7)	35 (53.8)	42 (50.6)	26 (47.3)		
Non-Hispanic Other	24 (8.5)	3 (3.8)	6 (9.2)	7 (8.4)	8 (14.5)		
Non-Hispanic White	48 (17.1)	15 (19.2)	14 (21.5)	11 (13.3)	8 (14.5)		
**Born in United States, No. (%)**							
Yes	194 (69.0)	57 (73.1)	49 (75.4)	53 (63.9)	35 (63.6)	0.31	0.0
No	87 (31.0)	21 (26.9)	16 (24.6)	30 (36.1)	20 (36.4)		
**English at home, No. (%)**							
Yes	262 (93.9)	74 (94.9)	62 (96.9)	76 (92.7)	50 (90.9)	0.53	0.7
No	17 (6.1)	4 (5.1)	2 (3.1)	6 (7.3)	5 (9.1)		
**Employment Status, No. (%)[b]**							
Employed	159 (56.8)	39 (50.6)	28 (43.1)	59 (71.1)	33 (60.0)	<0.01	0.4
Unemployed	121 (43.2)	38 (49.4)	37 (56.9)	24 (28.9)	22 (40.0)		
**Marital Status, No. (%)[c]**							
Married or in a marriage-like relationship	158 (56.2)	43 (55.1)	41 (63.1)	48 (57.8)	26 (47.3)	0.37	0.0
Single/Never Married/Separated/ Divorced/Widowed	123 (43.8)	35 (44.9)	24 (36.9)	35 (42.2)	29 (52.7)		
**Educational Attainment, No. (%)[d]**							
High School Graduate or Less	58 (20.6)	16 (20.5)	18 (27.7)	14 (16.9)	10 (18.2)	0.41	0.0
More than High School	223 (79.4)	62 (79.5)	47 (72.3)	69 (83.1)	45 (81.8)		
**Depression Risk (CES-D-SF ≥ 8, No. (%)[e]**							
Yes	68 (24.3)	15 (19.2)	17 (26.2)	20 (24.1)	16 (29.6)	0.56	0.4
No	212 (75.7)	63 (80.8)	48 (73.8)	63 (75.9)	38 (70.4)		
**Clinical and Behavioral Measures**							
**Height, mean (SD), centimeter**	172.8 (8.8)	173.5 (9.3)	172.0 (8.8)	174.1 (7.8)	170.9 (9.1)	0.14	0.0
**Weight, mean (SD), kilogram**	99.9 (18.2)	98.8 (18.4)	99.3 (18.4)	102.2 (14.1)	98.4 (22.8)	0.57	0.0

© 2025 Wittleder S et al. *JAMA Network Open*

Characteristic	Overall (N=281)	EUC: 52+ (n=78)	PAL: 52+ (n=65)	EUC: < 52 (n=83)	PAL: < 52 (n=55)	*p* value	*Missing (%)*
**BMI, mean (SD), kg/m^2**	33.4 (5.1)	32.9 (5.1)	33.5 (4.9)	33.7 (4.2)	33.6 (6.4)	0.81	0.0
**Waist circumference, mean (SD), in**	42.8 (5.4)	43.6 (4.9)	44.2 (5.7)	41.9 (4.3)	41.3 (6.5)	0.01	0.0
**Diastolic blood pressure, mean (SD), mmHg**	78.5 (19.9)	77.0 (11.5)	80.9 (37.2)	78.6 (9.5)	77.5 (10.0)	0.67	0.7
**Systolic blood pressure, mean (SD), mmHg**	125.6 (33.1)	126.2 (17.2)	126.8 (14.2)	127.6 (56.0)	120.2 (14.0)	0.60	0.7
**Walking Physical Activity, mean (SD), minutes/week[f]**	350.6 (383.3)	351.3 (373.0)	313.1 (335.4)	376.9 (440.7)	354.3 (364.1)	0.80	0.0
**Moderate Physical Activity, mean (SD), minutes/week[f]**	177.1 (278.3)	177.9 (278.2)	151.5 (248.7)	192.4 (286.1)	183.5 (304.9)	0.85	0.7
**Vigorous Physical Activity, mean (SD), minutes/week[f]**	204.0 (284.8)	198.6 (293.9)	175.9 (291.9)	226.8 (272.2)	210.4 (286.3)	0.75	0.0
**Total Physical Activity, mean (SD), MET minutes/week[f]**	3511.0 (3570.1)	3459.4 (3649.8)	3046.8 (3095.8)	3863.7 (3757.1)	3608.6 (3726.5)	0.58	0.7
**Fruit and Vegetable Intake, mean (SD)[g]**	2.4 (1.3)	2.3 (1.2)	2.5 (1.1)	2.3 (1.4)	2.7 (1.3)	0.14	0.0
**Healthy Dietary Behaviors, mean (SD)[h]**	13.2 (3.4)	12.9 (3.6)	13.2 (3.7)	13.3 (3.4)	13.6 (2.9)	0.74	0.4
**Sweet and Salty Snack Intake, mean (SD)[i]**	4.5 (1.2)	4.5 (1.1)	4.2 (1.2)	4.7 (1.2)	4.6 (1.0)	0.07	0.0
**Use of weight management programs and PAL**							
**Weight Management Program Attendance, %**							
Any	55 (26.8)	14 (20.9)	17 (37.8)	8 (13.3)	16 (48.5)	<0.01	27.0
None	150 (73.2)	53 (79.1)	28 (62.2)	52 (86.7)	17 (51.5)		
**Coaching calls completed, mean (SD)**	4.4 (3.0)	-	4.9 (3.0)	-	3.8 (3.0)	0.05	0.0

Abbreviations: BMI, body mass index; CES-D-SF, Center for Epidemiologic Studies Depression Scale Short-Form; EUC, enhanced usual care; mmHg, millimeters of mercury; MET, metabolic equivalent; PAL = Peer Assisted Lifestyle; SMD, standardized mean difference

[a] Race and ethnicity were self-reported by participants; Other races and ethnicities (n=7), Asian (n=6), American Indian or Alaskan Native (n=8), Native-Hawaiian/Pacific Islander (n=3) were included in the other category because of small cell sizes.

[b] Employed included working full time (n=144) and working part time (n=15). Unemployed included unemployed or laid off (n=14), looking for work (n=5), student (n=19), keeping house or raising children full-time (n=4), and retired (n=66).

[c] Marital Status included Single/Never Married (n=79), Married or in a marriage-like relationship (n=123), Separate (n=15), Divorced (n=59), and Widowed (n=5).

[d] Education Status included Never attended school or only kindergarten (n = 0), Grades 1 through 8 (Elementary) (n = 1), Grades 9 through 11 (n = 0), Grade 12 or GED (n = 57), Associate’s Degree (n = 48), Some college (n = 64), College 4 years or more (n = 71), Some graduate or professional training (n = 8), and Graduate or professional degree (n = 30).

[e] CES-D-SF is a seven-item screener for suspected major depressive disorder in US community studies (cutoff score ≥8).

© 2025 Wittleder S et al. *JAMA Network Open*

[f] We truncated walking, moderate, and vigorous durations to maximal 180 minutes and set days with < 10 minutes of activity to 0, consistent with the Physical Activity Questionnaire-Short Form scoring protocol. MET minutes were computed, representing the multiplicative value of energy expended carrying out physical activity >1 MET expended at rest (i.e., 3.3 for walking, 4 for moderate activity, and 8 for vigorous activity). We summed walking, moderate, and vigorous MET-minutes/week into a continuous total MET-minutes/week score.
[g] Fruit and Vegetable Intake was assessed using the six-item subscale of the Food Behavior Checklist. Scores could range from 1-4, with higher numbers indicating more/healthier fruits and vegetables intake.
[h] Healthy Dietary Behaviors were assessed using the six-item subscale from the Latino Dietary Behaviors Questionnaire. Scores could range from 0-24, with higher numbers indicating healthier eating behaviors.
[i] Sweet and Salty Snack Intake was assessed using two items from the Rapid Eating Assessment–Shortened Version. Scores could range from 2-6, with higher scores indicating healthier (less frequent) snack intake.

eTable 4. Sensitivity analyses of clinical outcomes at 6 and 12 months by treatment arm (excluding patients using obesity medications)

Outcomes[a,c]	EUC	PAL	Difference (PAL – EUC)	P-value	95% CI for difference (PAL – EUC)[b]
**Weight change (kg), 6 mo.**	-0.44 (0.40)	-2.09 (0.58)	-1.64 (0.69)	0.018	(-3.01, -0.28)
**Weight change (kg), 12 mo. [primary outcome]**	-0.79 (0.48)	-2.56 (0.74)	-1.77 (0.89)	0.048	(-3.52, -0.01)
**Weight change (%), 6 mo.**	-0.43 (0.39)	-1.90 (0.59)	-1.46 (0.70)	0.038	(-2.84, -0.08)
**Weight change (%), 12 mo.**	-0.69 (0.47)	-2.35 (0.77)	-1.66 (0.91)	0.068	(-3.45, 0.12)
**% with Weight Loss ≥5%, 6 mo.**	5.52 (3.17)	16.79 (4.79)	11.27 (5.26)	0.033	(0.92, 21.62)
**% with Weight Loss ≥5%, 12 mo.**	15.30 (3.40)	26.83 (5.21)	11.53 (6.27)	0.067	(-0.79, 23.84)
**Waist circumference change (in.), 6 mo.**	-0.72 (0.23)	-1.43 (0.32)	-0.71 (0.39)	0.072	(-1.48, 0.06)
**Waist circumference change (in.), 12 mo.**	-1.40 (0.29)	-1.83 (0.38)	-0.43 (0.49)	0.377	(-1.39, 0.53)

Abbreviations: CI, confidence interval; EUC, enhanced usual care; PAL, Peer Assisted Lifestyle.

[a] Values are adjusted means (standard errors).

[b] 95% confidence intervals for testing whether the difference in outcomes between the treatment arms are equal to zero are provided.

[c] Two patients (both assigned to the peer coaching arm) who started obesity care medication post-enrollment were excluded. No patients underwent bariatric surgery.

© 2025 Wittleder S et al. *JAMA Network Open*

eTable 5. Sensitivity analyses of behavioral outcomes at 6 and 12 months by treatment arm (excluding patients using obesity medications)

Outcomes[a,h]	EUC	PAL	Difference (PAL – EUC)	P-value	95% CI for difference (PAL – EUC)[b]
**Weight Management Program Attendance[c], %, 6 mo.**	13.38 (3.37)	27.99 (5.40)	-14.60 (6.49)	0.025	(1.86, 27.34)
**Weight Management Program Attendance[c], %. 12 mo.**	14.54 (3.75)	22.73 (4.42)	-8.19 (5.79)	0.157	(-3.16, 19.54)
**Change in Vigorous Physical Activity[d], min./week, 6 mo.**	49.95 (30.57)	75.90 (40.78)	-25.95 (52.09)	0.619	(-76.28, 128.18)
**Change in Vigorous Physical Activity[d], min./week 12 mo.**	36.36 (31.70)	0.78 (36.26)	35.58 (48.38)	0.462	(-130.49, 59.33)
**Change in Moderate Physical Activity[d], min./week, 6 mo.**	50.44 (27.54)	18.04 (33.50)	32.40 (44.48)	0.466	(-119.62, 54.81)
**Change in Moderate Physical Activity[d], min./week, 12 mo.**	8.86 (28.26)	13.42 (34.82)	-4.56 (45.65)	0.92	(-84.95, 94.07)
**Change in Walking Physical Activity[d], min./week, 6 mo.**	17.76 (39.71)	102.74 (49.81)	-84.98 (65.90)	0.197	(-44.27, 214.24)
**Change in Walking Activity[d], min./week, 12 mo.**	-27.38 (38.43)	-2.39 (46.24)	-24.99 (61.01)	0.682	(-94.62, 144.60)
**Change in Total Physical Activity[d], MET min./week, 6 mo.**	672.29 (328.49)	1029.42 (445.75)	-357.13 (572.08)	0.533	(-765.09, 1479.35)
**Change in Total Physical Activity[d], MET min./week, 12 mo.**	240.05 (328.63)	59.37 (386.05)	180.68 (517.51)	0.727	(-1195.49, 834.12)
**Change in Fruit and Vegetable intake[e], 6 mo.**	-0.14 (0.12)	-0.11 (0.14)	-0.03 (0.19)	0.878	(-0.34, 0.39)
**Change in Fruit and Vegetable intake[e], 12 mo.**	-0.32 (0.12)	-0.35 (0.14)	0.03 (0.19)	0.889	(-0.39, 0.34)
**Change in Healthy Dietary Behaviors[f], 6 mo.**	0.99 (0.58)	1.28 (0.69)	-0.29 (0.82)	0.724	(-1.32, 1.90)
**Change in Healthy Dietary Behaviors[f], 12 mo.**	1.48 (0.59)	1.91 (0.71)	-0.43 (0.84)	0.608	(-1.22, 2.08)
**Change in Sweet and Salty Snack Intake[g], 6 mo.**	0.24 (0.10)	0.62 (0.12)	-0.38 (0.16)	0.017	(0.07, 0.69)
**Change in Sweet and Salty Snack Intake[g], 12 mo.**	0.20 (0.11)	0.62 (0.14)	-0.42 (0.18)	0.018	(0.073, 0.77)

Abbreviations: CI, confidence interval; EUC, enhanced usual care; PAL, Peer Assisted Lifestyle; MET, metabolic equivalent; min., minutes.
[a] Values are adjusted means (standard errors).

© 2025 Wittleder S et al. *JAMA Network Open*

[b] 95% confidence intervals for testing whether the difference in outcomes between the treatment arms are equal to zero are provided.
[c] Weight management program attendance included both MOVE! and other weight management programs outside the VA (e.g. Weight Watchers). Weight management program attendance most often reported was the VA's MOVE! Program or the VA's Telephone Lifestyle Coaching at 6 month (n=32, 74.4%) and at 12 month (n=29, 74.4%).
[d] From the International Physical Activity Questionnaire – short form (IPAQ-SF), MET minutes were computed, representing the multiplicative value of energy expended carrying out physical activity > 1 MET expended at rest (i.e., 3.3 for walking, 4 for moderate activity, and 8 for vigorous activity). We summed walking, moderate, and vigorous MET-minutes/week into a continuous total MET-minutes/week score.
[e] Fruit and Vegetable Intake was assessed using the six-item subscale of the Food Behavior Checklist. Scores could range from 1-4, with higher numbers indicating more/healthier fruits and vegetables intake.
[f] Healthy Dietary Behaviors were assessed using the six-item subscale from the Latino Dietary Behaviors Questionnaire. Scores could range from 0-24, with higher numbers indicating healthier eating behaviors.
[g] Sweet and Salty Snack Intake was assessed using two items from the Rapid Eating Assessment–Shortened Version. Scores could range from 2-6, with higher scores indicating healthier (less frequent) snack intake.

[h] Two patients (both assigned to the peer coaching arm) who started obesity care medication post-enrollment were excluded. No patients underwent bariatric surgery.

© 2025 Wittleder S et al. *JAMA Network Open*

eReferences

1. Grundy SM, Brewer HB, Jr., Cleeman JI, et al. Definition of metabolic syndrome: report of the National Heart, Lung, and Blood Institute/American Heart Association conference on scientific issues related to definition. *Arterioscler Thromb Vasc Biol*. Feb 2004;24(2):e13-8. doi:10.1161/01.ATV.0000111245.75752.C6
2. Centre for Collaboration Motivation & Innovation. Accessed September 30, 2024. https://centrecmi.ca/
3. Gutnick D RK, Davis C, Gainforth H, Jay M, Cole S. Brief Action Planning to Facilitate Behavior Change and Support Patient Self-Management. *Journal of Clinical Outcomes Management*. 2014;21(1):17-29. doi:https://cmicourse.com/wp-content/uploads/JCOM-BAP-Evidence-paper-pdf-2014-1-20.pdf
4. Damschroder LJ, Goodrich DE, Kim HM, et al. Development and validation of the ASPIRE-VA coaching fidelity checklist (ACFC): a tool to help ensure delivery of high-quality weight management interventions. *Transl Behav Med*. Sep 2016;6(3):369-85. doi:10.1007/s13142-015-0336-x
5. Craig CL, Marshall AL, Sjostrom M, et al. International physical activity questionnaire: 12-country reliability and validity. *Med Sci Sports Exerc*. Aug 2003;35(8):1381-95. doi:10.1249/01.MSS.0000078924.61453.FB
6. International Physical Activity Questionnaire - Short Form. Accessed October 15, 2024. https://youthrex.com/wp-content/uploads/2019/10/IPAQ-TM.pdf
7. Wolin KY, Heil DP, Askew S, Matthews CE, Bennett GG. Validation of the International Physical Activity Questionnaire-Short among Blacks. *J Phys Act Health*. Sep 2008;5(5):746-60. doi:10.1123/jpah.5.5.746
8. Townsend MS, Kaiser LL, Allen LH, Joy AB, Murphy SP. Selecting items for a food behavior checklist for a limited-resource audience. *J Nutr Educ Behav*. Mar-Apr 2003;35(2):69-77. doi:10.1016/s1499-4046(06)60043-2
9. Kristal AR, Abrams BF, Thornquist MD, et al. Development and validation of a food use checklist for evaluation of community nutrition interventions. *Am J Public Health*. Nov 1990;80(11):1318-22. doi:10.2105/ajph.80.11.1318
10. Fernandez S, Olendzki B, Rosal MC. A dietary behaviors measure for use with low-income, Spanish-speaking Caribbean Latinos with type 2 diabetes: the Latino Dietary Behaviors Questionnaire. *J Am Diet Assoc*. Apr 2011;111(4):589-99. doi:10.1016/j.jada.2011.01.015
11. Segal-Isaacson CJ, Wylie-Rosett J, Gans KM. Validation of a short dietary assessment questionnaire: the Rapid Eating and Activity Assessment for Participants short version (REAP-S). *Diabetes Educ*. Sep-Oct 2004;30(5):774, 776, 778 passim. doi:10.1177/014572170403000512
12. Johnston CS, Bliss C, Knurick JR, Scholtz C. Rapid Eating Assessment for Participants [shortened version] scores are associated with Healthy Eating Index-2010 scores and other indices of diet quality in healthy adult omnivores and vegetarians. *Nutr J*. Sep 28 2018;17(1):89. doi:10.1186/s12937-018-0399-x
13. van Breukelen GJ, Candel MJ. Calculating sample sizes for cluster randomized trials: we can keep it simple and efficient! *J Clin Epidemiol*. Nov 2012;65(11):1212-8. doi:10.1016/j.jclinepi.2012.06.002
14. Damschroder LJ, Lutes LD, Kirsh S, et al. Small-Changes Obesity Treatment Among Veterans 12-Month Outcomes. *American Journal of Preventive Medicine*. Nov 2014;47(5):541-553. doi:10.1016/j.amepre.2014.06.016
15. Levine DM, Savarimuthu S, Squires A, Nicholson J, Jay M. Technology-assisted weight loss interventions in primary care: a systematic review. *J Gen Intern Med*. Jan 2015;30(1):107-17. doi:10.1007/s11606-014-2987-6

© 2025 Wittleder S et al. *JAMA Network Open*
