# Supplementary material for: Peer Coaching to Support Weight Management in Primary Care: A Cluster Randomized Clinical Trial
Source: JAMA Netw Open. 2025 Sep 2;8(9):e2529136. doi: 10.1001/jamanetworkopen.2025.29136 (PMC12406063; doi:10.1001/jamanetworkopen.2025.29136)
Supplement: Supplement 3. — Data Sharing Statement [file jamanetwopen-e2529136-s003.pdf]

Data Sharing Statement

Wittleder. Peer Coaching to Support Weight Management in Primary Care. *JAMA Netw Open*. Published September 02, 2025. doi:10.1001/jamanetworkopen.2025.29136

Data
Additional Information: The Peer Assisted Lifestyle Intervention, https://clinicaltrials.gov/study/NCT03163264?intr=peer%20assisted%20lifestyle&rank=2, Trial Registration: NCT03163264
Data available: Yes
Data types: Deidentified participant data
How to access data: Request de-identified by contacting corresponding author. A de-identified dataset and data dictionary will be made available on a publicly available US data repository pending approval by the funding sponsor. The study protocol and statistical analysis plan are available at https://www.clinicaltrials.gov.
When available: With publication

Supporting Documents
Document types: None

Additional Information
Who can access the data: Researchers whose proposed use of the data has been approved
Types of analyses: For a specified purpose
Mechanisms of data availability: After approval of a proposal, data will be sent via email initially and eventually via a data repository.
